# Effects of high-intensity interval training versus moderate-intensity continuous training on vascular function among individuals with overweight and obesity—a systematic review

**DOI:** 10.1038/s41366-024-01586-4

**Published:** 2024-07-30

**Authors:** Shishira K B, K. Vaishali, Rajagopal Kadavigere, Suresh Sukumar, Shivashankara K N, Samuel A. Pullinger, Tulasiram Bommasamudram

**Affiliations:** 1 Department of Physiotherapy, Manipal College of Health Professions (MCHP), Manipal Academy of Higher Education (MAHE), Manipal, 576104 India; 2 Department of Radiodiagnosis and Imaging, Kasturba Medical College (KMC), Manipal Academy of Higher Education (MAHE), Manipal, 576104 India; 3 Department of Medical Imaging Technology, Manipal College of Health Professions (MCHP), Manipal Academy of Higher Education (MAHE), Manipal, 576104 India; 4 Department of Medicine, Kasturba Medical College (KMC), Manipal Academy of Higher Education (MAHE), Manipal, Karnataka 576104 India; 5 Sport Science Department, Inspire Institute of Sport, Vidyanagar, District Bellary, 583275 India; 6 Department of Exercise and Sports Sciences, Manipal College of Health Professions (MCHP), Manipal Academy of Higher Education (MAHE), Manipal, Karnataka 576104 India

**Keywords:** Lifestyle modification, Weight management

## Abstract

**Background:**

The study aimed to investigate and systematically review the evidence relating to the effects of high-intensity interval training (HIIT) versus moderate-intensity continuous training (MICT) on vascular function such as arterial diameter, arterial stiffness, pulse wave velocity, blood flow, etc. in individuals with overweight and obesity.

**Methods:**

The entire content of PubMed (MEDLINE), Scopus, SPORT Discus® (via EBSCO host), CINAHL, and Web of Science were searched. Only experimental research studies conducted in adult participants aged ≥18 years, published in English before January 2023 were included.

**Results:**

A total of 5397 studies were reviewed for the title and abstract with 11 studies being included for data extraction. The review resulted in a total of 346 individuals with overweight and obesity with body mass index (BMI) ranging between 25–36 kg/m^2^. HIIT and MICT intensities resulted in 85%–95% and 60%–70% maximal heart rate (MHR) respectively. Seven out of 11 studies showed some concerns about the overall risk of bias. Six of 11 studies reported improving vascular function following HIIT than MICT.

**Conclusion:**

HIIT is a more effective and time-efficient exercise for enhancing vascular functions in individuals with overweight and obesity, leading to improvements in flow-mediated dilation by 3.9% and arterial diameter by 4.8%, compared to MICT.

## Introduction

Globally, the prevalence of individuals with overweight and obesity, defined by a body mass index (BMI) of 25 or higher, is steadily increasing, presenting a significant health threat linked to both morbidity and mortality [1, 2]. The condition contributes significantly to the development of non-communicable diseases, with obesity and being overweight acting as notable risk factors. The occurrence of vascular dysfunction is significantly more common in adults who are overweight or with obesity compared to those of with normal weight. Research shows that individuals with obesity exhibit a 54–57% decrease in flow-mediated dilation (FMD), indicating vascular dysfunction [3, 4]. This condition is a major risk factor for cardiovascular diseases, including hypertension, coronary artery disease, and stroke, metabolic abnormalities, including dyslipidemia, hypertension, oxidative stress, insulin resistance, and heightened inflammation [5, 6]. These factors increase the risk of vascular dysfunction by diminishing nitric oxide bioavailability and disrupting vascular homeostasis [5].

The term “vascular function” refers to the ability of the vessel to expand in response to stimulation or shear stress, achieved through the heightened synthesis of nitric oxide. This fundamental process serves as the cornerstone for vessel relaxation and vasodilation, crucial elements in maintaining optimal blood flow and overall cardiovascular health [7, 8]. The assessment of vascular function plays a pivotal role in understanding the intricate dynamics of the circulatory system and can be assessed invasively or non-invasively. Non-invasive techniques are particularly essential, providing a comprehensive understanding of vascular health without the risks associated with invasive procedures. Approaches such as Doppler ultrasound, flow-mediated dilation (FMD), arterial stiffness measurement (e.g.: Pulse Wave Velocity (PWV), Augmentation Index (AIx), Ankle-Brachial Index (ABI)), carotid intima-media thickness (cIMT) and arterial tonometry offer valuable insights into the efficiency and responsiveness of blood vessels [9]. These methods contribute significantly to our understanding of vascular health, enabling a nuanced exploration of the factors influencing circulatory dynamics.

Exercise is one of the therapeutic interventions that has shown significant improvement in cardiovascular health, particularly in enhancing vascular health [10]. It has demonstrated the ability to increase nitric oxide availability, improve endothelial function, reduce oxidative stress, and aid in vascular remodeling [10–14]. Moderate Intensity Continuous Training (MICT) has been extensively studied. It involves prolonged periods of activity at 40–80% of maximal oxygen consumption and has proven effective in reducing the risk of cardiovascular disease [15]. Another form of exercise known as high-intensity interval training (HIIT) entails brief and intense intervals of activity interspersed with periods of rest or low-intensity exercise and has gained interest in recent years [16]. Remarkably, HIIT has been shown to improve fitness and health among young adults with overweight and obesity [17, 18]. However, current literature presents mixed findings regarding the comparative effectiveness of HIIT and MICT in improving vascular function. Some studies suggest that HIIT is more effective than MICT in enhancing FMD in individuals with obesity, indicating better vascular adaptations due to the high-intensity bursts of activity in HIIT [19–21]. This suggests that HIIT may stimulate more significant improvements in endothelial function compared to the steady, moderate exercise of MICT. Conversely, other studies report no significant difference between HIIT and MICT as FMD remained unchanged [22]. This inconsistency extends to other measures of vascular function, such as arterial diameter, blood flow, and velocity. For instance, some research indicates that both HIIT and MICT produce similar changes in arterial diameter and flow, while others find no measurable changes at all, regardless of the exercise regimen [23]. Despite these contradictory findings, studies suggest that HIIT can yield comparable, if not superior, improvements in cardiorespiratory fitness, insulin sensitivity, and endothelial function compared to standard MICT [20, 21]. This suggests that while the specific effects on vascular function may vary, HIIT generally offers substantial cardiovascular and metabolic benefits. These benefits are particularly pronounced in populations with obesity, where HIIT has shown promise in improving several key health markers more efficiently than traditional moderate-intensity exercises. This highlights the complexities and varying outcomes associated with different exercise regimens, underlying the need for further research to fully understand the distinct benefits and limitations of HIIT and MICT on vascular health.

Previous reviews have consistently affirmed that HIIT is a more effective strategy for enhancing vascular function than MICT. However, these conclusions are predominantly based on studies that have concentrated on populations such as those with coronary artery disease (CAD), hypertension, metabolic syndrome, congestive heart failure (CHF), and so on [21, 24, 25]. These studies have contributed valuable insights into the impact of HIIT and MICT programs on vascular function. Notably, a comprehensive systematic study conducted by Way et al. [26] arrived at a distinct conclusion, stating that no significant differences were observed in central arterial stiffness between HIIT and MICT. This diversity in results underlines the ongoing uncertainty regarding the most effective exercise modality for enhancing vascular function in individuals with overweight and obesity. Therefore, this systematic review aims to investigate the influence of both HIIT and MICT on vascular function among individuals with overweight or obesity.

## Methods

### Eligibility criteria

This systematic review has adhered to the Preferred Reporting Items for Systematic Reviews and Meta-Analyses (PRISMA) guidelines [27]. The inclusion criteria were developed using the Cochrane guidelines for conducting systematic reviews [28]. All the authors decided upon and approved the inclusion and exclusion criteria. Following the original selection of studies, the eligibility assessment was carried out independently, blindly, by two authors (SKB and TB) who screened the abstracts and titles. The following requirements had to be met for the manuscript to be considered for inclusion:

1. Population—adults (18–60 years) who are overweight or with obesity, with a BMI of 25 kg/m^2^ and above without any health issues such as CVD, neurological disorders, diabetes, hypertension, metabolic disorders, musculoskeletal injuries, and sleep apnea.
2. Intervention—high-intensity interval training, which alternates between periods of low-intensity exercise or rest and short bursts of extremely intense activity, such as running, cycling, or workouts with a VO_2max_ or MHR more than 80% of intensity and moderate-intensity interval training involving prolonged periods of activity of walking or cycling with 40–80% of VO_2max_ both given for a minimum period of 4 weeks.
3. Comparison—high-intensity interval training versus moderate-intensity interval training. Studies that involve interventions other than HIIT and MICT or those that include only one of these were excluded.
4. Outcome variables—assessing vascular functions such as endothelial dysfunction, arterial stiffness, flow-mediated dilation, and pulse wave velocity.
5. Design—randomized trials or non-randomized trials like quasi-experimental, pre-post study involving the human population and published in English before January 2023 were included. Other study designs such as cross-over trials, case reports, case-control, cross-sectional designs, animal studies, letters to the editor, conference abstracts, and literature reviews were excluded.

### Literature search strategy and information sources

**Fig. 1:**
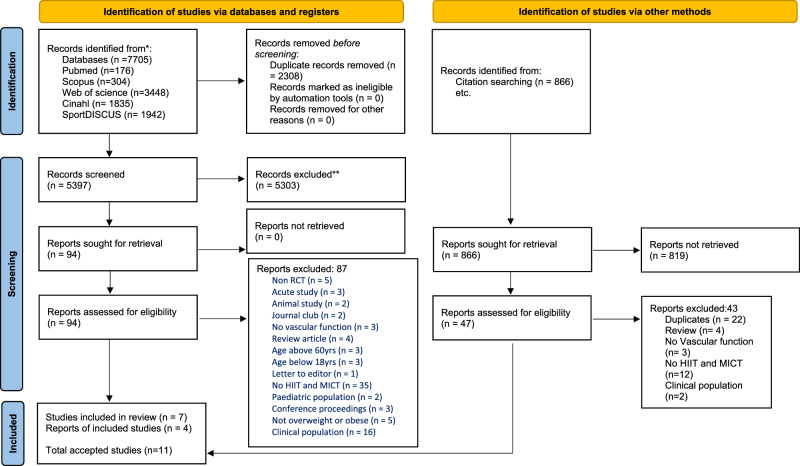
PRISMA 2020 flow diagram indicating the number of studies retained and excluded at each stage of the review process. PRISMA 2020 flow diagram for new systematic reviews which included searches of databases, registers, and other sources.

The computerized English-language literature search was conducted using the Manipal Academy of Higher Education electronic library, PubMed (MEDLINE), Scopus, SPORT Discus® (via EBSCOhost), CINAHL, and Web of Science electronic databases. To find pertinent data on HIIT, MICT, and vascular function in the titles, abstracts, and keywords of the indexed publications, the following search syntax was used with Boolean operators: (“high-intensity interval training”[Mesh] OR “high intensity interval training” OR HIIT) OR (“aerobic interval training” OR “continuous training” OR “moderate-intensity continuous exercise” OR MICT OR “endurance” OR “walking” OR “running” OR “cycling”) AND (“obesity” OR “overweight”) AND (“Vascular Stiffness” OR “Blood Flow Velocity” OR “endothelial function” OR “vascular function” OR “vascular resistance” OR “flow-mediated dilation” OR “peak wave velocity” OR “pulse wave velocity” OR “intima-media thickness” OR “nitric oxide” OR “Arter* complian*” OR “arter* elasticit*” OR “vascular* stiff*” OR “vascular* complian*” OR “vascular* elasticit*”) (Appendix 1). Furthermore, truncation, proximity searching, and other sophisticated search techniques were included. Covidence software was used to manually screen the articles and reference lists of all included papers for additional relevant publications (SKB & TB) as part of the secondary search. In order to investigate the possibility of leveraging authors and citations for follow-up investigations, forward reference searching was also done. To mitigate potential selection bias, TB carried out the study selection searches on their own. The flow of articles by the study selection process is shown in Fig. 1 using the PRISMA 2020 flow diagram [27].

### Study selection

When the title and abstract of an article were not sufficient to evaluate its relevance to the review, the complete text of the article was retrieved and reviewed. This allowed the writers to determine if the work satisfied the primary inclusion conditions. If the primary goal of the publications did not entail a study looking at how HIIT and MICT affect vascular function, they were excluded from the review.

### Data extraction

The two reviewers (SKB and TB) retrieved data independently, while a third (KV) checked the data with the following data extracted from the included papers: (1) the study authors and date; (2) the number of participants and participant’s characteristics (age, gender, body weight, and BMI); (3) exercise protocol (frequency, intensity, time, and type of HIIT and MICT); (4) outcome measure; (5) equipment used; (6) main findings—vascular function variables which were assessed.

### Quality assessment

The quality of all randomized studies was assessed using the Risk of Bias (ROB) 2.0 tool that addresses five domains of the randomization process, deviation from intended intervention, missing outcome data, measurement of the outcome, and selection of the reported result. The quality of the non-randomized study was assessed using the Risk of Bias in Non-randomized Studies—of Interventions (ROBINS-I) tool by the recommendation of the Cochrane Scientific Committee. This tool addresses 7 domains namely: confounding, selection of participants, classification of interventions, deviations from intended interventions, missing data, measurement of outcomes, and selection of the reported result. Three categories—“high,” “some concerns,” and “low” risk of bias—are used to group these assessments that are based on the responses to the signaling questions in both tools to determine the overall bias judgment. A visualization of the outcomes was produced using “traffic light” plots of the domain-level judgments for each result, following the guidelines provided by each evaluation tool.

## Results

### Search results

The initial database search yielded 7705 articles, and a further 866 via citation and organization searches. The number of articles discovered in each electronic database or by using other techniques is shown in Fig. 1, along with a comprehensive flowchart of the literature search’s phases. After duplicates were eliminated, 5397 titles collected from databases were still included in the Covidence software. Following a review of all the papers’ titles, abstracts, and keywords, 94 papers were sought for retrieval and retained for full-text analysis. The systematic review included 7 articles after determining that the inclusion criteria were eligible, and 87 articles were excluded. Figure 1 shows the explanations for exclusion. The eligibility of a total of reports found through organizations and citation searching was evaluated, and of these 4 were included in the review.

### Study characteristics

A detailed study characteristics of eleven studies that were included in this review are shown in Table 1, the publication year of these studies ranged from 2008 to 2023. Ten of the included studies were randomized trials and one resulted in a quasi-experimental study. The review incorporated three studies focusing on individuals with overweight [22, 23, 29], five studies on individuals with obesity [19, 30–33], and three studies involving individuals with both overweight and obesity [34–36]. Eight of these studies were supervised training [19, 22, 23, 31, 33–36] whereas three of them were a combination of supervised and unsupervised training [29, 30, 32].

**Table 1:** Summary of the articles reviewed for individuals with overweight and obesity (*n* = 11) with an overview of the participant characteristics, exercise protocol, exercise supervision, outcome variables, equipment used, and the main findings related to vascular functions.

Author	Participants characteristics	Exercise protocol		Exercise supervision	Outcome variable	Equipment used	Main findings
		HIIT	MICT				
Farahati et al. [36]	33 women who are inactive and overweight	85%–95% HR_max_, 4 × 4 min, 3 S WK^−1^, 12 WK	60%–70% HR_max_ Treadmill Walking, 47 min, 3 S WK^−1^, 12 WK	Supervised	Carotid IMT (mm)	Doppler ultrasound, M-Turbo, Sonosite America	MICT ↔ HIIT ↔ control ↔
	HIIT (*n* = 10); 42.8 ± 2.69 yrs; 29.2 ± 2.28 kg/m²				Overall ABI	Doppler ultrasound, M-Turbo, Sonosite America	MICT ↔ HIIT ↔ control ↔
	MICT (*n* = 11); 43.9 ± 3.8 yrs; 30.79 ± 2.79 kg/m²				Right ABI	Doppler ultrasound, M-Turbo, Sonosite America	MICT↔ HIIT↑15%* control ↔
	Control (*n* = 9); 44.22 ± 3.63 yrs; 31.63 ± 3.97 kg/m²				Left ABI	Doppler ultrasound, M-Turbo, Sonosite America	MICT↔ HIIT↔ Control↔
Tucker et al. [35]	28 men who are overweight and obese	90%–95% HR_max_ Cycling, 8–11 × 1 min, 4 S W^−1^, 4 WK	50% VO_2_ max cycling, 30–40 min, 4 S W^−1^, 4 WK	Supervised	FMD	Terason t3000CV Ultrasound, Terason, Burlington, MA	HIIT↔ MICT↔ CON↔
	HIIT (*n* = 10); 30 ± 7 yrs; 96.0 ± 7.7 kg; 30.2 ± 3 kg/m²				Baseline diameter	Terason t3000CV Ultrasound, Terason, Burlington, MA	HIIT↔ MICT↔ CON↔
	MICT (*n* = 9); 29 ± 7 yrs; 94.5 ± 18.6 kg; 29.7 ± 4.5 kg/m²				Peak diameter	Terason t3000CV Ultrasound, Terason, Burlington, MA	HIIT↔ MICT↔ CON↔
	Control (*n* = 9); 28 ± 9 yrs; 93.1 ± 11.8 kg; 29.6 ± 3.9 kg/m²				Peak shear stress	Terason t3000CV Ultrasound, Terason, Burlington, MA	HIIT↔ MICT↔ CON↔
					Mean blood velocity	Terason t3000CV Ultrasound, Terason, Burlington, MA	HIIT↔ MICT↔ CON↔
Baekkerud et al. [34]	30 overweight and obese			Supervised	Venous compliance		4HIIT↔ 1 HIIT↔ MICT↔
	4HIIT (*n* = 12); 39 ± 10 yr; 91.7 ± 17.4 kg; BMI, 31.4 ± 5.3 kg/m²	85%–95% HR_max_ Treadmill walking, 4 × 4 min, 3 S WK^−1^, 6 WK	70% HR_max_ Steady state running/walking, 45 min, 3 S WK^−1^, 6 WK		Peak venous outflow	14-MHz Doppler probe, Vivid 7 System; GE Vingmed Ultrasound, Horten, Norway	4HIIT↔ 1 HIIT↔ MICT↓22.7%
	1HIIT (*n* = 9); 45 ± 8 yr; 91.1 ± 10.1 kg; 30.8 ± kg/m²	90% HR_max_, 10 × 1 min, 3 S WK^−1^, 6 WK			Arterial inflow	14-MHz Doppler probe, Vivid 7 System; GE Vingmed Ultrasound, Horten, Norway	4HIIT↔ 1 HIIT↔ MICT↓ 15.7%
	MICT (*n* = 9) 41 ± 10 yr; 89.5 ± 12.4 kg; 29 ± 2.7 kg/m²				FMD	14-MHz Doppler probe, Vivid 7 System; GE Vingmed Ultrasound, Horten, Norway	4HIIT↔ 1 HIIT↔ MICT↔
Shepherd et al. [29]	90 individuals who are healthy, inactive and overweight	>90% HRmax Sprints, 15–60 s, 18–25 min, 5 S WK^−1^, 10 WK	~70% HRmax Cycling, 30 min (WK 1)–45 min (WK 10), 5 S WK^−1^, 10 WK	3 supervised 2 unsupervised	AIx@75bpm	–	HIIT↔ MICT↔
	HIT (*n* = 46); 42 ± 11 yrs; 78.8 ± 18.3 kgs; 27.7 ± 5.0 kg/m²						
	MICT (*n* = 44); 43 ± 11 yrs; 77.5 ± 15.8 kgs; 27.7 ± 4.6 kg/m²						
Shenouda et al. [23]	27 men who are sedentary, overweight	All out cycling sprints against 5.0% body weight, 3× 20 s, 30 S, 12 WK	70% HRmax Cycling, 45 min, 30 S, 12 WK	Supervised	Brachial artery diameter (BAD)	Artery Measurement System (AMS) II, version 1.141; Gothenburg, Sweden	Resting BAD HIIT↑0.5% MICT↑8% Control ↑3%
	HIIT (*n* = 9); 27 ± 7 yrs; 84 ± 23 kg; 27 ± 5 kg/m²				FMD	Doppler ultrasounds (Vivid Q; GE Medical Systems, Horten, Norway)	HIIT↔ MICT↔ CNT ↔
	MICT (*n* = 10); 28 ± 9 yrs; 84 ± 20 kg; 26 ± 6 kg/m²				Popliteal artery diameter	Artery Measurement System (AMS) II, version 1.141; Gothenburg, Sweden	HIIT↔ MICT↔ CNT↔
	Control (*n* = 6); 26 ± 8 yrs; 78 ± 25 kg; 25 ± 7 kg/m²				FMD	Doppler ultrasounds (Vivid Q; GE Medical Systems, Horten, Norway)	HIIT↔ MICT↔ CNT↔
					Carotid distensibility	Doppler ultrasound (Vivid Q, GE Medical Systems) & applanation tonometry (model SPT-301, Millar Instruments, Houston, TX)	HIIT↔ MICT↔ CNT↔
					PWV	Applanation tonometry (Mikro-Tip Catheter Transducer, model SPT-301; Millar Instruments)	HIIT↔ MICT↔ CNT↔
Chin et al. [22]	56 men who are overweight; 18–30 yrs; 26.4 ± 2.9 kg/m²						
	HIIT 3 (*n* = 14)	90% HRR 30-m Shuttle run, 12 × 1 min, 3 S WK^−1^, 8 WK	60% HRR running, 30 min, 3 S WK^−1^, 8 WK	Supervised	LnRHI	EndoPAT 2000 (ITAMAR Medical, Caesarea, Israel)	HIIT 3↔ HIIT2↔ HIIT 1↔ MICT↔
	HIIT 2 (*n* = 10)	90% HRR 30-m Shuttle run, 12 × 1 min, 2 S WK^−1^, 8 WK			AIx@75	EndoPAT 2000 (ITAMAR Medical, Caesarea, Israel)	HIIT 3↔ HIIT2↔ HIIT 1↔ MICT↔
	HIIT 1 (*n* = 9)	90% HRR 30-m Shuttle run, 12 × 1 min, 1 S WK^−1^, 8 WK					
	MICT (*n* = 9)						
	Control (*n* = 14)						
Cheema et al. [30]	12 adults with BMI >25 kg/m²	>75% HRmax, RPE of 15-17/20 Boxing, 4 S × 50 min WK^−1^, 12 WK	Brisk Walking, 4 S × 50 min WK^−1^, 12 WK	HIIT: Supervised MICT: unsupervised	AIx	SphygmoCor System (AtCor Medical Pty, Sydney, Australia)	HIIT ↓126.7%* MICT ↔
	HIIT (*n* = 6); 43 yrs; 95.7 kg; 32.0 kg/m²				Pulse pressure	Fidelity tonometer (Millar Instruments, Houston, Texas)	HIIT ↔ MICT ↔
	MICT (*n* = 6); 36 yrs; 85.6 kg; 30.8 kg/m²						
Cocks et al. [31]	16 men who are sedentary and obese	200% Wmax Cycling, 1–3 S 4 reps, ↑1 rep every 3 S, 3 S WK^−1^, 4 WK	~65% VO2 peak Cycling, 1–7 S 40 min, 8–14 S 50 min, 15–20 S 60 min, 5 S WK^−1^, 4 WK	Supervised	AIx	SphygmoCor, AtCor Medical, Sydney, Australia	HIIT↓* MICT↓*
	HIIT (*n* = 8); 24 ± 2 yrs; 110 ± 5 kg; 35.8 ± 0.8 kg/m²				cPWV	SphygmoCor, AtCor Medical, Sydney, Australia	HIIT↓* MICT↓*
	MICT (*n* = 8); 26 ± 2 yrs; 113 ± 6 kg; 33.7 ± 1.5 kg/m²				pPWV	SphygmoCor, AtCor Medical, Sydney, Australia	HIIT↔ MICT↔
Sawyer et al. [19]	18 adults who are obese M(18–45 yrs); F(18–55 yrs)	90%–95% HRmax Cycling, 10 × 1-min, 24 S:3 S WK^−1^, 8 WK	70–75% HRmax Cycling, 30 min, 24 S:3 S WK^−1^, 8 WK	Supervised	Preocclusion baseline diameter	Terason t3000 high resolution (Terason Ultrasound, Burlington, MA)	HIIT ↔ MICT ↑4.9%*
	HIIT (*n* =9); 37.4 ± 6.2 kg/m²				Postocclusion peak diameter	Terason t3000 high resolution (Terason Ultrasound, Burlington, MA)	HIIT ↑5%* MICT ↔
	MICT (*n* = 9); 34.5 ± 3.2 kg/m²						
					Flow-mediated dilation	Terason t3000 high resolution (Terason Ultrasound, Burlington, MA)	HIIT ↑*3.8% MICT ↔
					Shear rate area under the curve	Terason t3000 high resolution (Terason Ultrasound, Burlington, MA)	HIIT ↓28%* MICT ↔
					Average velocity, cm/s	Terason t3000 high resolution (Terason Ultrasound, Burlington, MA)	HIIT ↓23%* MICT ↔
					Average diameter, mm	Terason t3000 high resolution (Terason Ultrasound, Burlington, MA)	HIIT ↑4.8%* MICT ↔
Schjerve et al. [32]	40 adults who are obese	85%–95% HRmax, 4 × 4 min, 3 S WK^−1^, 12 WK	60–70% HRmax Walking, 40 min, 3 S WK^−1^, 12 WK	2 supervised 1 unsupervised	Flow-mediated dilation	High-resolution vascular ultrasound 14MHz echo Doppler probe; Vivid 7 system; GE Vingmed Ultrasound	HIIT greater ↑* MICT↑* ST↑*
	HIIT (*n* = 14); 46.9 ± 2.2 yrs; 114.0 ± 5.7 kg; 36.6 ± 1.2 kg/m²				BA baseline diameter		HIIT↔ MICT↔ ST↔
	MICT (*n* = 13); 44.4 ± 2.1 yrs; 104.1 ± 4.5 kg; 36.7 ± 1.4 kg/m²				BA Shear rate		HIIT↔ MICT↔ ST↔
	ST (*n* = 13); 46.2 ± 2.9 yrs; 98.8 ± 4.5 kg; 34.5 ± 1.4 kg/m²	90% 1RM leg press, 4 × 5 reps, Abdominal and Back Ex 3 × 30 reps, 3 S WK^−1^, 12 WK			Peak NTG diameter		HIIT↔ MICT↔ ST↔
Shi et al. [33]	52 adults who are obese 18–22 years, ≥30 kg/m²	80%–95% HRmax Ex, WK 1–2: 40 s, 25 min Ex, WK 3–5: 60 s 35 min Ex, WK 6–8: 60 s 45 min EX, 3–4 S WK^−1^, 8 WK	60%–70% HRmax Ex, 45 min, 3–4 S WK^−1^, 8 WK	Supervised	Blood volume	Doppler ultrasound (Prosound Alpha 7, Aloka, Japan)	HIIT ↑11.5%* MICT ↑18.1%* CON ↔
	HIIT (*n* = 21)				Flow rate	Doppler ultrasound (Prosound Alpha 7, Aloka, Japan)	HIIT↔ MICT↔ CON↔
	MICT (*n* = 22)				Wall shear stress	Doppler ultrasound (Prosound Alpha 7, Aloka, Japan)	HIIT ↑21.6%* MICT ↑16.9%* CON ↔
	Control (*n* = 9)				OSI	Doppler ultrasound (Prosound Alpha 7, Aloka, Japan)	HIIT ↓* MICT ↔ CON ↔
					Arterial diameter	Doppler ultrasound (Prosound Alpha 7, Aloka, Japan)	HIIT ↓6%* MICT ↔ CON ↔
					Arterial stiffness	Doppler ultrasound (Prosound Alpha 7, Aloka, Japan)	HIIT ↓18.8%* MICT ↔ CON ↔
					Circumferential strain	Doppler ultrasound (Prosound Alpha 7, Aloka, Japan)	HIIT ↑18.1%* MICT ↔ CON ↔
					Pulsatility index	Doppler ultrasound (Prosound Alpha 7, Aloka, Japan)	HIIT ↓7%* MICT ↔ CON ↔

↓ decrease, ↔ no change, ↑ increase, * significant, *S* session, *WK* week, *s* seconds, *min* minutes, *yrs* years, *kg/m*^*2*^ kilogram per meter square (BMI), *1 RM* 1 repetition maximum, *BA* brachial artery, *AIx* augmentation index, *AIx@75bpm* augmentation index at 75 beats per minute, *cPWV* central pulse wave velocity, *pPWV* peripheral pulse wave velocity, *IMT* intima-media thickness, *ABI* ankle-brachial index, *BA* brachial artery, *NTG* nitroglycerin, *OSI* oscillatory index, *FMD* flow-mediated dilation, *LnRHI* natural log of the reactive hyperemia index, *HIIT* high-intensity interval training, *MICT* moderate-intensity continuous training.

### Participant characteristics

The detailed characteristics of the participants in the included studies are presented in Table 1. A total of 346 participants with an average of 31 participants per study were included. The number of participants in each study ranged from 12 to 90. The age of the participants ranged from 18 to 55 years and the BMI of the participants ranged from 25 to 36 kg/m^2^. Both genders were included in 6 studies, 4 included only men and 1 included female participants only. In this review, healthy individuals with overweight and obesity were included.

### Intervention characteristics

The intervention characteristics of the included studies are described in Table 1. The frequency of the training ranged from 3 to 5 sessions per week with 3 sessions per week being the most common among 7 studies [19, 22, 29, 31, 32, 34, 36]. Most of the research used HIIT sessions that ranged in intensity from 85% to 95% of MHR; a small number of the studies utilized 90% Heart Rate Reserve (HRR) and active recovery periods. Two studies chose to use Sprint Interval Training (SIT), which is characterized by intense bursts of activity, in contrast to most of the included studies that used HIIT [23, 31]. MICT protocols included 60%–70% MHR and an HRR for a duration of 45 min that ranged between 30 min [19] to 60 min [31]. One study compared strength training with an intensity of 90% 1 RM along with HIIT and MICT [32]. The intervention duration ranged from 4 weeks [31] to 12 weeks, with 12 weeks being practiced in 4 studies [23, 30, 32, 36]. Most of the studies used cycling, treadmill walking, and running as the modality of intervention. Five studies included a control group among which four studies were advised to maintain their routine [22, 23, 35, 36] whereas in one study control group was educated about the dangers of obesity, its causes, and ways to improve through lectures and videos, and online communication groups [33].

### Measurement of vascular function

Vascular function among individuals with overweight and obesity was assessed in 11 studies, and significant improvements were observed in at least one of the assessed parameters, including FMD, venous compliance, Pulse Wave Velocity (PWV), and Augmentation Index (AIx) in eight of the studies. Focusing on the comparative effects of HIIT and MICT on FMD in five studies, two studies revealed a substantial enhancement in FMD by 3.8% following HIIT [19, 32]. Significant improvements in post-occlusion peak diameter (5%), mean artery diameter (23%), shear rate (28%), and mean blood flow velocity (23%) were also seen with HIIT, while these remained unchanged in MICT [19, 32]. Three additional studies found no significant alterations in FMD following either exercise modality [23, 34, 35]. Additionally, among six studies examining arterial stiffness, two reported significant improvements in AIx 126.7% [30, 31], one in PWV [31], and one in Ankle-Brachial Index (ABI) 15% [36] within the HIIT group. Conversely, two studies found no changes in AIx [22, 29], and one study found PWV and carotid distensibility remained unchanged [23]. Particularly, Shi et al. [33] noted significant improvements in Wall Shear Stress (21.6%), circumferential strain (18.1%), and a decrease in arterial stiffness (18.8%), pulsatility index (7%), arterial diameter (6%), and oscillatory shear index in the HIIT group [33]. These findings underline the varied impact of HIIT and MICT on vascular parameters.

### Methodological quality control and publication bias

**Fig. 2:**
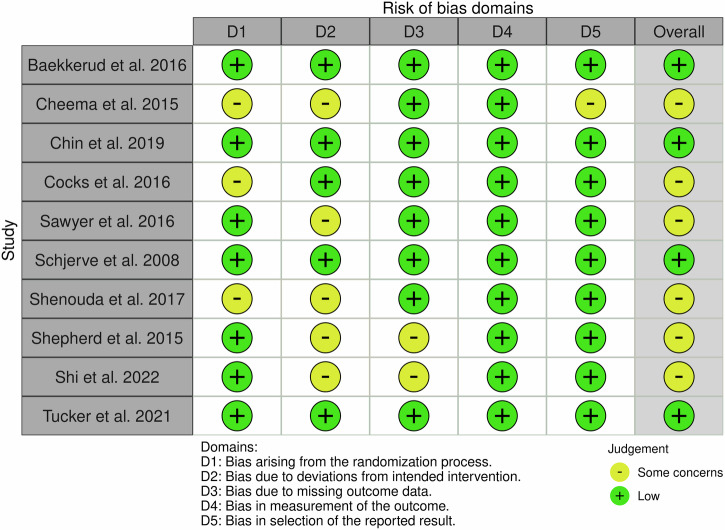
Risk-of-bias ViSualization (robvis). Risk of bias of the ten included studies, according to the RoB 2.0 tool using the “traffic light” plots of the domain-level judgments for each individual result [48].

**Fig. 3:**
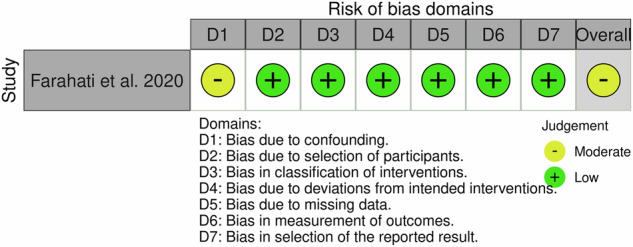
Risk-of-bias ViSualization (robvis). Risk of bias of one included study, according to the ROBINS-I tool using the “traffic light” plots of the domain-level judgments for each individual result [48].

The risk of bias (ROB) 2.0 tool was used in ten studies and the entire results are shown in Fig. 2. All ten studies in outcome measurement (Domain 4), nine studies in reported result selection (Domain 5), eight studies in missing outcome data (Domain 3), seven studies in randomization process (Domain 1), and five studies in deviation from intended intervention (Domain 2) have a low risk of bias. Whereas five studies in the domain of deviation from the intended intervention (Domain 2), three from the randomization method, two studies in missing outcome data (Domain 3), and one research in the domain of selection of reported outcomes (Domain 5) raise some concerns. The ROBINS tool was used in one non-randomized study [36] (Fig. 3). Across all domains, bias due to confounding (Domain 1) was discovered to have a moderate bias risk, whereas all other domains exhibited a low bias risk.

## Discussion

This study aimed to systematically review and compare the effects of high-intensity interval training (HIIT) and moderate-intensity continuous training (MICT) on various measures of vascular function in individuals with overweight and obesity. In this review, data from 11 trials were analyzed to determine the effects of HIIT and MICT interventions on vascular functioning. The following are the significant findings from this assessment: firstly, a considerable proportion of the publications (72%, *N* = 8) showed significant differences in at least one vascular function variable. Second, most studies found that HIIT interventions outperformed MICT in terms of improving vascular functioning.

In six studies, HIIT outperformed MICT, strength training, and control interventions in improving vascular function. A meta-analysis undertaken by Sabouri et al. [25] supports this corroborative tendency by highlighting HIIT as a particularly beneficial technique for increasing FMD in individuals dealing with overweight or obesity. This meta-analysis data suggests that HIIT increases FMD by 2.6% compared to MICT and by 1.83% compared to no exercise intervention [25]. Studies by Sawyer et al. [19] and Schjerve et al. [32] both demonstrate significant improvements in FMD—3.89% after 8 weeks and a noticeable improvement after 12 weeks of HIIT, respectively [19, 32]. Importantly, studies established that a mere 1% increase in FMD corresponded to an 8–13% decrease in the risk of cardiovascular disease [37]. Exercise-related increases in FMD may be caused by an increase in NO bioavailability brought on by increased blood flow and higher shear pressures on the endothelium [38]. Increased exercise training lowers pro-inflammatory and oxidative stress factors linked to vascular dysfunction [39]. Accordingly, HIIT’s superior ability to improve vascular function over MICT might result from its capacity to increase blood flow to working muscle, which facilitates higher NO bioavailability generated by shear stress [40, 41]. In line with this, Shi et al. [33] discovered that after 8 weeks of HIIT, there was a rise in blood flow and wall shear stress (11.5% and 21.6%) [33]. However, few studies showed that there were no differences in FMD between HIIT, MICT, and control groups [34, 35]. This could be attributed primarily to elevated baseline endothelial function, potentially impeding FMD improvement [34]. Additionally, some participants in these studies had consumed doughnuts, potentially hindering the anticipated improvements [35].

On the other hand, a study conducted by Shenouda et al. [23] demonstrated that 12 weeks of traditional MICT was more effective than HIIT in producing notable enhancements in brachial artery FMD and artery diameter, which were not observed in HIIT. The potentially pivotal factor behind this discrepancy could be the lower volume of HIIT compared to MICT, possibly contributing to the superior results observed with the latter [23].

Arterial stiffness was evaluated by five studies, Cheema et al. [30] observed a significant reduction in the augmentation index (AIx) after 12 weeks of high-intensity boxing sessions lasting 50 min each, accompanied by enhancements in endothelial function and peripheral resistance [30]. Shi et al. [33] reported a notable decrease in arterial stiffness with a higher circumferential strain and a lower oscillatory shear index following HIIT, contrasting with MICT [33]. Although not statistically significant, the improvement in arterial stiffness after HIIT may be attributed to an increase in circumferential strain, as it has been shown to negatively correlate with arterial stiffness [42]. Farahati et al. [36] discovered a significantly higher right Ankle-Brachial Index (ABI) in the HIIT group after 12 weeks of training at an intensity of 85%–95% of maximal heart rate [36]. These findings align with previous assessments demonstrating that aerobic exercise significantly reduces PWV and AIx in adults, as indicated by studies like Ashor et al. [43] and Huang et al. [43, 44]. Contrarily, Cocks et al. [31] research indicated that both HIIT and MICT significantly increased AIx and carotid-femoral Pulse Wave Velocity (cPWV) [31]. However, inconsistencies exist, as reflected in a systematic review by Way et al. [26] which found no discernible difference in changes to central arterial stiffness measured by AIx, AIx@75, and PWV between HIIT and MICT [26]. In accordance with this, Shepherd et al. [29] also reported no change in AIx after using either training mode [29].

When compared to MICT, HIIT is known to provide 40% gain in time efficiency [17, 45, 46]. The greatest hurdle to exercise is time, which prevents people from engaging in it regularly [47]. The time-effectiveness of HIIT improves the possibility of overcoming this barrier and encouraging better adherence to regular exercise. An intriguing aspect of this comparison is that both MICT and HIIT result in comparable levels of energy expenditure [32, 35]. This demonstrates the adaptability of HIIT, proving its ability to provide physiological benefits comparable to typical moderate-intensity exercise in significantly less time. The parallel energy expenditure strengthens the case for HIIT as a feasible alternative, particularly for persons with time constraints or wanting a more time-efficient workout schedule.

### Strengths and limitations

This systematic review’s strength is that it distinguishes itself through a robust methodology, adhering closely to the PRISMA 2020 guidelines. The strength of the review is enhanced by the comprehensive integration of a diverse range of databases in the search strategy, coupled with the meticulous application of specific search terms. Noteworthy is the stringent adherence to inclusion criteria, ensuring the inclusion of only those studies that evaluated HIIT and MICT while also assessing vascular function. However, it is crucial to acknowledge a limitation inherent in this systematic review, arising from substantial methodological differences. A notable difference is the exclusive use of MHR to determine HIIT intensity, without considering VO2max. Future studies should consider including VO2max to provide a more comprehensive exercise intensity assessment. Additionally, the diverse approaches employed in assessing vascular function across the 11 included studies further contribute to this limitation. These variabilities rendered it impractical to conduct a meta-analysis and amalgamate the observed datasets to comprehensively evaluate evidence regarding the impact of HIIT and MICT on vascular functioning. The impediment primarily emanated from the methodological and clinical heterogeneity inherent in these 11 studies, underscoring the need for cautious interpretation due to these intrinsic differences.

## Conclusion

The systematic review suggests that HIIT is more effective than MICT in improving vascular function (FMD, arterial stiffness, and wall shear stress) and can be a time-efficient exercise model. The findings align with prior studies showing a link between cardiovascular fitness and vascular function. Consistent with a recent meta-analysis, HIIT is highlighted for its superior ability to enhance cardiovascular fitness. Importantly, these benefits extend to low weekly exercise volumes, exhibit rapid manifestation, and remain consistent across diverse BMI statuses. Overall, HIIT emerges as a promising strategy for efficiently improving vascular function across various conditions.

## Appendix 1. Literature search strategy example for PubMed (MEDLINE)

**Search syntax**

(“high-intensity interval training”[Mesh] OR “high intensity interval training” OR HIIT OR “aerobic interval training” OR “continuous training” OR “moderate-intensity continuous exercise” OR MICT OR “endurance” OR “walking” OR “running” OR “cycling”)

AND

(“obesity” OR “overweight”)

AND

(“Vascular Stiffness” OR “Blood Flow Velocity” OR “endothelial function” OR “vascular function” OR “vascular resistance” OR “flow-mediated dilation” OR “peak wave velocity” OR “pulse wave velocity” OR “intima-media thickness” OR “nitric oxide” OR “Arter* complian*” OR “arter* elasticit*” OR “vascular* stiff*” OR “vascular* complian*” OR “vascular* elasticit*”)

**Records identified and screened**

***N*** **=** **176**

1. 30324108 Cardiovascular Effects and Benefits of Exercise Nystoriak MA, Bhatnagar A. Front Cardiovasc Med. 2018 Sep 28; 5:135. doi: 10.3389/fcvm.2018.00135. eCollection 2018. Nystoriak MA Front Cardiovasc Med 2018 17-10-2018 PMC6172294.
2. 31343521 Low-Frequency HIIT Improves Body Composition and Aerobic Capacity in Overweight Men Chin EC, Yu AP, Lai CW, Fong DY, Chan DK, Wong SH, Sun F, Ngai HH, Yung PSH, Siu PM. Med Sci Sports Exerc. 2020 Jan;52(1):56-66. doi: 10.1249/MSS.0000000000002097. Chin EC Med Sci Sports Exerc 2020 26-07-2019.
3. 31694237 Effects of Endurance and Endurance-Strength Training on Endothelial Function in Women with Obesity: A Randomized Trial Ratajczak M, Skrypnik D, Bogdański P, Mądry E, Walkowiak J, Szulińska M, Maciaszek J, Kręgielska-Narożna M, Karolkiewicz J. Int J Environ Res Public Health. 2019 Nov 5;16(21):4291. doi: 10.3390/ijerph16214291. Ratajczak M Int J Environ Res Public Health 2019 08-11-2019 PMC6862069.
4. 27885969 36th International Symposium on Intensive Care and Emergency Medicine: Brussels, Belgium. 15-18 March 2016 Bateman RM, Sharpe MD, Jagger JE, Ellis CG, Solé-Violán J, López-Rodríguez M, Herrera-Ramos E, Ruíz-Hernández J, Borderías L, Horcajada J, González-Quevedo N, Rajas O, Briones M, Rodríguez de Castro F, Rodríguez Gallego C, Esen F, Orhun G, Ergin Ozcan P, Senturk E, Ugur Yilmaz C, Orhan N, Arican N, Kaya M, Kucukerden M, Giris M, Akcan U, Bilgic Gazioglu S, Tuzun E, Riff R, Naamani O, Douvdevani A, Takegawa R, Yoshida H, Hirose T, Yamamoto N, Hagiya H, Ojima M, Akeda Y, Tasaki O, Tomono K, Shimazu T, Ono S, Kubo T, Suda S, Ueno T, Ikeda T, Hirose T, Ogura H, Takahashi H, Ojima M, Kang J, Nakamura Y, Kojima T, Shimazu T, Ikeda T, Suda S, Izutani Y, Ueno T, Ono S, Taniguchi T, O M, Dinter C, Lotz J, Eilers B, Wissmann C, Lott R, Meili MM, Schuetz PS, Hawa H, Sharshir M, Aburageila M, Salahuddin N, Chantziara V, Georgiou S, Tsimogianni A, Alexandropoulos P, Vassi A, Lagiou F, Valta M, Micha G, Chinou E, Michaloudis G, Kodaira A, Ikeda T, Ono S, Ueno T, Suda S, Izutani Y, Imaizumi H, De la Torre-Prados MV, Garcia-De la Torre A, Enguix-Armada A, Puerto-Morlan A, Perez-Valero V, Garcia-Alcantara A, Bolton N, Dudziak J, Bonney S, Tridente A, Nee P, et al. Crit Care. 2016 Apr 20;20(Suppl 2):94. doi: 10.1186/s13054-016-1208-6. Bateman RM Crit Care 2016 26-11-2016 PMC5493079.
5. 33106031 A Novel Endocrine Role for the BAT-Released Lipokine 12,13-diHOME to Mediate Cardiac Function Pinckard KM, Shettigar VK, Wright KR, Abay E, Baer LA, Vidal P, Dewal RS, Das D, Duarte-Sanmiguel S, Hernández-Saavedra D, Arts PJ, Lehnig AC, Bussberg V, Narain NR, Kiebish MA, Yi F, Sparks LM, Goodpaster BH, Smith SR, Pratley RE, Lewandowski ED, Raman SV, Wold LE, Gallego-Perez D, Coen PM, Ziolo MT, Stanford KI. Circulation. 2021 Jan 12;143(2):145-159. doi: 10.1161/CIRCULATIONAHA.120.049813. Epub 2020 Oct 27. Pinckard KM Circulation 2021 27-10-2020 PMC7856257 NIHMS1652325.
6. 33022918 Effects of Moderate Combined Resistance- and Aerobic-Exercise for 12 Weeks on Body Composition, Cardiometabolic Risk Factors, Blood Pressure, Arterial Stiffness, and Physical Functions, among Obese Older Men: A Pilot Study. Park W, Jung WS, Hong K, Kim YY, Kim SW, Park HY. Int J Environ Res Public Health. 2020 Oct 3;17(19):7233. doi: 10.3390/ijerph17197233. Park W Int J Environ Res Public Health 2020; 07-10-2020; PMC7579509.
7. 31963313 Cardiovascular Health Effects of Shift Work with Long Working Hours and Night Shifts: Study Protocol for a Three-Year Prospective Follow-Up Study on Industrial Workers Lunde LK, Skare Ø, Mamen A, Sirnes PA, Aass HCD, Øvstebø R, Goffeng E, Matre D, Nielsen P, Heglum HSA, Hammer SE, Skogstad M. Int J Environ Res Public Health. 2020 Jan 16;17(2):589. doi:10.3390/ijerph17020589. Lunde LK; Int J Environ Res Public Health 2020; 23-01-2020; PMC7014249.
8. 33760255 Low-volume high-intensity interval training for cardiometabolic health; Sabag A, Little JP, Johnson NA. J Physiol. 2022 Mar;600(5):1013-1026. doi: 10.1113/JP281210. Epub 2021 Apr 18. Sabag AJ Physiol 2022 24-03-2021.
9. 36121143 Hypoxic high-intensity interval training in individuals with overweight and obesity Ghaith A, Chacaroun S, Borowik A, Chatel L, Doutreleau S, Wuyam B, Tamisier R, Pépin JL, Flore P, Verges S. Am J Physiol Regul Integr Comp Physiol. 2022 Nov 1;323(5): R700-R709. doi: 10.1152/ajpregu.00049.2022. Epub 2022 Sep 19. Ghaith A; Am J Physiol Regul Integr Comp Physiol 2022 19-09-2022.
10. 37074396 Liraglutide and Exercise Synergistically Attenuate Vascular Inflammation and Enhance Metabolic Insulin Action in Early Diet-Induced Obesity; Liu J, Aylor KW, Liu Z. Diabetes. 2023 Jul 1;72(7):918-931. doi: 10.2337/db22-0745. Liu J Diabetes 2023 19-04-2023 PMC10281235.
11. 35499629 Effect of exercise training on arterial stiffness in obese and overweight children: a meta-analysis Cheng Y, Sun Z, Ya X, Zhou L, Wang M, Wang X, Liu Y. Eur J Pediatr. 2022 Jul;181(7):2633-2642. doi: 10.1007/s00431-022-04489-6. Epub 2022 Apr 30. Cheng Y Eur J Pediatr 2022 02-05-2022.
12. 37203832 Effect of high intensity interval training on arterial stiffness in obese hypertensive women: a randomized controlled trial Taha MM, Aneis YM, Hasanin ME, Felaya EE, Aldhahi MI, Abdeen HAA. Eur Rev Med Pharmacol Sci. 2023 May;27(9):4069-4079. doi: 10.26355/eurrev_202305_32314. Taha MM Eur Rev Med Pharmacol Sci 2023 19-05-2023.
13. 35468651 Effect of Exercise Training on Arterial Stiffness in Overweight or Obese Populations Gong L, Liu Y. Int J Sports Med. 2022 Nov;43(12):996-1012. doi: 10.1055/a-1795-2940. Epub 2022 Apr 25. Gong L Int J Sports Med 2022 25-04-2022 PMC9622304.
14. 26666335 Causes of changes in carotid intima-media thickness: a literature review; Qu B, Qu T. Cardiovasc Ultrasound. 2015 Dec 15; 13:46. doi: 10.1186/s12947-015-0041-4. Qu B Cardiovasc Ultrasound: 2015 16-12-2015 PMC4678459.
15. 26931178 Exercise training improves obesity-related lymphatic dysfunction Hespe GE, Kataru RP, Savetsky IL, García Nores GD, Torrisi JS, Nitti MD, Gardenier JC, Zhou J, Yu JZ, Jones LW, Mehrara BJ. J Physiol. 2016 Aug 1;594(15):4267-82. doi: 10.1113/JP271757. Epub 2016 Apr 9. Hespe GE J Physiol 2016 03-03-2016 PMC4967732.
16. 37345828 Interleukin-1β Disruption Protects Male Mice From Heart Failure With Preserved Ejection Fraction Pathogenesis Srinivas BK, Bourdi A, O'Regan JD, Malavalli KD, Rhaleb NE, Belmadani S, Matrougui K. J Am Heart Assoc. 2023 Jul 18;12(14):e029668. doi: 10.1161/JAHA.122.029668. Epub 2023 Jun 22. Srinivas BK J Am Heart Assoc 2023 22-06-2023 PMC10382083.
17. 32555019 Vascular Protection by Exercise in Obesity: Inflammasome-associated Mechanisms Lee J, Hong J, Umetani M, Lavoy EC, Kim JH, Park Y. Med Sci Sports Exerc. 2020 Dec;52(12):2538-2545. doi: 10.1249/MSS.0000000000002419. Lee J Med Sci Sports Exerc 2020 20-06-2020.
18. 35806938 Cardiorespiratory Fitness and Carotid Intima-Media Thickness in Physically Active Young Adults: CHIEF Atherosclerosis Study Lin GM, Liu PY, Tsai KZ, Lin YK, Huang WC, Lavie CJ. J Clin Med. 2022 Jun 24;11(13):3653. doi: 10.3390/jcm11133653. Lin GM J Clin Med 2022 09-07-2022 PMC9267611.
19. 31552710 Endothelial function following interval exercise plus low-calorie diet treatment in obese females Gilbertson NM, Miller SL, Eichner NZM, Malin SK. Physiol Rep. 2019 Sep;7(18): e14239. doi: 10.14814/phy2.14239. Gilbertson NM Physiol Rep 2019 26-09-2019 PMC6759506.
20. 32102058 Hypoxic Exercise Training to Improve Exercise Capacity in Obese Individuals Chacaroun S, Borowik A, Vega-Escamilla Y Gonzalez I, Doutreleau S, Wuyam B, Belaidi E, Tamisier R, Pepin JL, Flore P, Verges S. Med Sci Sports Exerc. 2020 Aug;52(8):1641-1649. doi: 10.1249/MSS.0000000000002322. Chacaroun S Med Sci Sports Exerc 2020 27-02-2020.
21. 28256934 Clinical impact of exercise in patients with peripheral arterial disease. Novakovic M, Jug B, Lenasi H. Vascular. 2017 Aug;25(4):412-422. doi:10.1177/1708538116678752. Epub 2016 Nov 9. Novakovic M Vascular 2017 04-03-2017.
22. 34923497 Endurance Exercise Training-Attenuated Diabetic Kidney Disease with Muscle Weakness in Spontaneously Diabetic Torii Fatty Rats Kotake H, Yamada S, Ogura Y, Watanabe S, Inoue K, Ichikawa D, Sugaya T, Ohata K, Natsuki Y, Hoshino S, Watanabe M, Kimura K, Shibagaki Y, Kamijo-Ikemori A. Kidney Blood Press Res. 2022;47(3):203-218. doi: 10.1159/000521464. Epub 2021 Dec 17. Kotake H Kidney Blood Press Res 2022 19-12-2021.
23. 29099231 Effects of High-Intensity Intermittent Training on Vascular Function in Obese Preadolescent Boys Chuensiri N, Suksom D, Tanaka H. Child Obes. 2018 Jan;14(1):41-49. doi: 10.1089/chi.2017.0024. Epub 2017 Nov 3. Chuensiri N Child Obes 2018 04-11-2017.
24. 35324646 Vascular Function in Norwegian Female Elite Runners: A Cross-Sectional, Controlled Study Kyte KH, Stensrud T, Berg TJ, Seljeflot I, Hisdal J. Sports (Basel). 2022 Mar 2;10(3):37. doi: 10.3390/sports10030037. Kyte KH Sports (Basel) 2022 24-03-2022 PMC8955513.
25. 35109880 Effects of high-intensity interval training on improving arterial stiffness in Chinese female university students with normal weight obese: a pilot randomized controlled trial Hu J, Liu M, Yang R, Wang L, Liang L, Yang Y, Jia S, Chen R, Liu Q, Ren Y, Zhu L, Cai M. J Transl Med. 2022 Feb 2;20(1):60. doi: 10.1186/s12967-022-03250-9. Hu JJ Transl Med 2022 03-02-2022 PMC8809004.
26. 36729923 Effect of Acute Walking on Endothelial Function and Postprandial Lipemia in South Asians and White Europeans Roberts MJ, Thackray AE, Wadley AJ, Alotaibi TF, Hunter DJ, Thompson J, Fujihira K, Miyashita M, Mastana S, Bishop NC, O'Donnell E, Davies MJ, King JA, Yates T, Webb D, Stensel DJ. Med Sci Sports Exerc. 2023 May 1;55(5):794-802. doi: 10.1249/MSS.0000000000003098. Epub 2022 Dec 5. Roberts M J Med Sci Sports Exerc 2023 02-02-2023 PMC10090289.
27. 32053417 Cardiorespiratory and metabolic responses to exercise testing during lower-body positive pressure running Brüssau T, Oehring R, Felix SB, Dörr M, Bahls M. J Appl Physiol (1985). 2020 Apr 1;128(4):778-784. doi:10.1152/japplphysiol.00328.2019. Epub 2020 Feb 13. Brüssau T J Appl Physiol (1985) 2020 14-02-2020.
28. 35357089 Hepatocyte-specific eNOS deletion impairs exercise-induced adaptations in hepatic mitochondrial function and autophagy Cunningham RP, Moore MP, Dashek RJ, Meers GM, Jepkemoi V, Takahashi T, Vieira-Potter VJ, Kanaley JA, Booth FW, Rector RS. Obesity (Silver Spring). 2022 May;30(5):1066-1078. doi: 10.1002/oby.23414. Epub 2022 Mar 31. Cunningham RP Obesity (Silver Spring) 2022 31-03-2022 PMC9050943NIHMS1783738.
29. 36192605 High-intensity interval training ameliorates endothelial dysfunction through adropin, nitric oxide, MR-proADM, and copeptin changes in overweight subjects zad Hormones (Athens). 2022 Dec;21(4):707-717. doi: 10.1007/s42000-022-00402-w. Epub 2022 Oct 4. Abbasian S Hormones (Athens) 2022 03-10-2022.
30. 36981579 Comparison of Hemodynamic and Cerebral Oxygenation Responses during Exercise between Normal-Weight and Overweight Men Wang SH, Lin HL, Huang CC, Chen YH. Healthcare (Basel). 2023 Mar 22;11(6):923. doi: 10.3390/healthcare11060923. Wang SH Healthcare (Basel) 2023 29-03-2023 PMC10048205.
31. 28803380 Aerobic interval training reduces vascular resistances during submaximal exercise in obese metabolic syndrome individuals; Mora-Rodriguez R, Fernandez-Elias VE, Morales-Palomo F, Pallares JG, Ramirez-Jimenez M, Ortega JF. Eur J Appl Physiol. 2017 Oct;117(10):2065-2073. doi: 10.1007/s00421-017-3697-7. Epub 2017 Aug 12. Mora-Rodriguez R Eur J Appl Physiol 2017 14-08-2017.
32. 25642686 Acute exercise effects on vascular and autonomic function in overweight men; Chandrakumar D, Boutcher SH, Boutcher YN. J Sports Med Phys Fitness. 2015 Jan-Feb;55(1-2):91-102. Chandrakumar DJ Sports Med Phys Fitness 2015 03-02-2015.
33. 35401768 Effects of high intensity interval training on sustained reduction in cardiometabolic risk associated with overweight/obesity. A randomized trial Mendelson M, Chacaroun S, Baillieul S, Doutreleau S, Guinot M, Wuyam B, Tamisier R, Pépin JL, Estève F, Tessier D, Vergès S, Flore P. J Exerc Sci Fit. 2022 Apr;20(2):172-181. doi: 10.1016/j.jesf.2022.03.001. Epub 2022 Mar 19.m Mendelson MJ Exerc Sci Fit 2022 11-04-2022 PMC8956941.
34. 33315809 Endurance and Sprint Training Improve Glycemia and V˙O2 peak but only Frequent Endurance Benefits Blood Pressure and Lipidemia Petrick HL, King TJ, Pignanelli C, Vanderlinde TE, Cohen JN, Holloway GP, Burr JF. Med Sci Sports Exerc. 2021 Jun 1;53(6):1194-1205. doi: 10.1249/MSS.0000000000002582. Petrick HL Med Sci Sports Exerc 2021 14-12-2020.
35. 37674777 The effect of aerobic exercise training on asthma control in postmenopausal women (ATOM): a randomized controlled pilot study; Hansen ESH, Rasmusen HK, Hostrup M, Hellsten Y, Backer V. Eur Clin Respir J. 2023 Sep 4;10(1):2251256. doi: 10.1080/20018525.2023.2251256. eCollection 2023. Hansen ESH Eur Clin Respir J 2023 07-09-2023 PMC10478610.
36. 27572153 Regular Exercise Reduces Endothelial Cortical Stiffness in Western Diet-Fed Female Mice Padilla J, Ramirez-Perez FI, Habibi J, Bostick B, Aroor AR, Hayden MR, Jia G, Garro M, DeMarco VG, Manrique C, Booth FW, Martinez-Lemus LA, Sowers JR. Hypertension. 2016 Nov;68(5):1236-1244. doi: 10.1161/HYPERTENSIONAHA.116.07954. Epub 2016 Aug 29. Padilla J Hypertension 2016 31-08-2016 PMC5063712NIHMS808832.
37. 31603262 The effect of interval sprinting exercise on vascular function and aerobic fitness of post-menopausal women Ho TY, Redmayne GP, Tran A, Liu D, Butlin M, Avolio A, Boutcher SH, Boutcher YN. Scand J Med Sci Sports. 2020 Feb;30(2):312-321. doi: 10.1111/sms.13574. Epub 2019 Nov 5. Ho TY Scand J Med Sci Sports 2020 12-10-2019.
38. 11583105 Exercise prescription for the elderly: current recommendations Mazzeo RS, Tanaka H. Sports Med. 2001;31(11):809-18. doi: 10.2165/00007256-200131110-00003. Mazzeo RS Sports Med 2001 05-10-2001.
39. 31609300 Interval Exercise Lowers Circulating CD105 Extracellular Vesicles in Prediabetes Eichner NZM, Gilbertson NM, Heiston EM, Musante L, LA Salvia S, Weltman A, Erdbrugger U, Malin SK. Med Sci Sports Exerc. 2020 Mar;52(3):729-735. doi: 10.1249/MSS.0000000000002185. Eichner NZM Med Sci Sports Exerc 2020 15-10-2019 PMC7024030NIHMS1540872.
40. 25809076 The effect of different training modes on skeletal muscle microvascular density and endothelial enzymes controlling NO availability; Cocks M, Wagenmakers AJ. J Physiol. 2016 Apr 15;594(8):2245-57. doi:10.1113/JP270329. Epub 2015 Apr 16. Cocks MJ Physiol 2016 27-03-2015 PMC493.
41. 24561866 Exercise performance and peripheral vascular insufficiency improve with AMPK activation in high-fat diet-fed mice Baltgalvis KA, White K, Li W, Claypool MD, Lang W, Alcantara R, Singh BK, Friera AM, McLaughlin J, Hansen D, McCaughey K, Nguyen H, Smith IJ, Godinez G, Shaw SJ, Goff D, Singh R, Markovtsov V, Sun TQ, Jenkins Y, Uy G, Li Y, Pan A, Gururaja T, Lau D, Park G, Hitoshi Y, Payan DG, Kinsella TM. Am J Physiol Heart Circ Physiol. 2014 Apr 15;306(8):H1128-45. doi: 10.1152/ajpheart.00839.2013. Epub 2014 Feb 21. Baltgalvis KA Am J Physiol Heart Circ Physiol 2014 25-02-2014 PMC3989755.
42. 31338769 High-intensity interval training combined with resistance training improved apnea-hypopnea index but did not modify oxygen desaturation index and oxygen saturation nadir in obese children with obstructive sleep apnea; Longlalerng K, Sonsuwan N, Uthaikhup S, Kietwatanachareon S, Kamsaiyai W, Panyasak D, Pratanaphon S. Sleep Breath. 2020 Jun;24(2):571-580. doi: 10.1007/s11325-019-01899-z. Epub 2019 Jul 23. Longlalerng K Sleep Breath 2020 25-07-2019.
43. 22439679 Exercise training and impaired glucose tolerance in obese humans McNeilly AM, McClean C, Murphy M, McEneny J, Trinick T, Burke G, Duly E, McLaughlin J, Davison G. J Sports Sci. 2012;30(8):725-32. doi: 10.1080/02640414.2012.671952. Epub 2012 Mar 23. McNeilly AM J Sports Sci 2012 24-03-2012.
44. 20093665 Strength training versus aerobic interval training to modify risk factors of metabolic syndrome: Stensvold D, Tjønna AE, Skaug EA, Aspenes S, Stølen T, Wisløff U, Slørdahl SA. J Appl Physiol (1985). 2010 Apr;108(4):804-10. doi: 10.1152/japplphysiol.00996.2009. Epub 2010 Jan 21. Stensvold DJ Appl Physiol (1985) 2010 23-01-2010.
45. 32755410 Pregnancy Protects Hyperandrogenemic Female Rats From Postmenopausal Hypertension Shawky NM, Patil CN, Dalmasso C, Maranon RO, Romero DG, Drummond H, Reckelhoff JF. Hypertension. 2020 Sep;76(3):943-952. doi: 10.1161/HYPERTENSIONAHA.120.15504. Epub 2020 Aug 3. Shawky NM Hypertension 2020 07-08-2020 PMC7429272 NIHMS1608287.
46. 30281094 High-Intensity Interval Training Improves Aerobic Capacity Without a Detrimental Decline in Blood Glucose in People with Type 1 Diabetes. Scott SN, Cocks M, Andrews RC, Narendran P, Purewal TS, Cuthbertson DJ, Wagenmakers AJM, Shepherd SO. J Clin Endocrinol Metab. 2019 Feb 1;104(2):604-612. doi: 10.1210/jc.2018-01309. Scott SN J Clin Endocrinol Metab 2019 04-10-2018.
47. 36657899 High-intensity interval training improves the vascular endothelial function comparing moderate-intensity interval training in overweight or obese adults: A meta-analysis. Sabouri M, Amirshaghaghi F, Hesari MM. Clin Nutr ESPEN. 2023 Feb; 53:100-106. doi: 10.1016/j.clnesp.2022.11.023. Epub 2022 Dec 7. Sabouri M Clin Nutr ESPEN 2023 19-01-2023.
48. 353614421 Year HIIT and Omega-3 Fatty Acids to Improve Cardiometabolic Risk in Stage-A Heart Failure Hearon CM Jr, Dias KA, MacNamara JP, Hieda M, Mantha Y, Harada R, Samels M, Morris M, Szczepaniak LS, Levine BD, Sarma S. JACC Heart Fail. 2022 Apr;10(4):238-249. doi: 10.1016/j.jchf.2022.01.004. Epub 2022 Mar 9. Hearon CM Jr JACC Heart Fail. 2022 01-04-2022.
49. 18338980 Both aerobic endurance and strength training programs improve cardiovascular health in obese adults Schjerve IE, Tyldum GA, Tjønna AE, Stølen T, Loennechen JP, Hansen HE, Haram PM, Heinrich G, Bye A, Najjar SM, Smith GL, Slørdahl SA, Kemi OJ, Wisløff U. Clin Sci (Lond). 2008 Nov;115(9):283-93. doi: 10.1042/CS20070332. Schjerve IE Clin Sci (Lond) 2008 15-03-2008.
50. 33638689 Impact of acute high-intensity interval exercise on plasma pentraxin 3 and endothelial function in obese individuals-a pilot study Slusher AL, Fico BG, Dodge KM, Garten RS, Ferrandi PJ, Rodriguez AA, Pena G, Huang CJ. Eur J Appl Physiol. 2021 Jun;121(6):1567-1577. doi: 10.1007/s00421-021-04632-5. Epub 2021 Feb 27. Slusher AL Eur J Appl Physiol 2021 27-02-2021.
51. 32534655 Impact of obesity on walking capacity and cardiovascular parameters in patients with peripheral artery disease: A cross-sectional study Kanegusuku H, Cucato GG, Domiciano RM, Longano P, Puech-Leao P, Wolosker N, Ritti-Dias RM, Correia MA. J Vasc Nurs. 2020 Jun;38(2):66-71. doi: 10.1016/j.jvn.2020.02.004. Epub 2020 Mar 26. Kanegusuku HJ Vasc Nurs 2020 15-06-2020.
52. 19897059 Biochemical effects of SIRT1 activators Baur JA. Biochim Biophys Acta. 2010 Aug;1804(8):1626-34. doi: 10.1016/j.bbapap.2009.10.025. Epub 2009 Nov 6. Baur JA Biochim Biophys Acta 2010 10-11-2009 PMC2886178NIHMS158173.
53. 29747089 Effects of high-intensity interval training on optic nerve head and macular perfusion using optical coherence tomography angiography in healthy adults Schmitz B, Nelis P, Rolfes F, Alnawaiseh M, Klose A, Krüger M, Eter N, Brand SM, Alten F. Atherosclerosis. 2018 Jul; 274:8-15. doi: 10.1016/j.atherosclerosis. 2018.04.028. Epub 2018 Apr 26. Schmitz B Atherosclerosis 2018 11-05-2018.
54. 37680774 Cardiorespiratory fitness and development of childhood cardiovascular risk: The EXAMIN YOUTH follow-up study Hauser C, Lichtenstein E, Nebiker L, Streese L, Köchli S, Infanger D, Faude O, Hanssen H. Front Physiol. 2023 Aug 23; 14:1243434. doi: 10.3389/fphys.2023.1243434. eCollection 2023. Hauser C Front Physiol 2023 08-09-2023 PMC10482095.
55. 32444343 The effect of acute aerobic exercise on central arterial stiffness, wave reflections, and hemodynamics in adults with diabetes: A randomized cross-over design Way KL, Lee AS, Twigg SM, Johnson NA. J Sport Health Sci. 2021 Jul;10(4):499-506. doi: 10.1016/j.jshs.2020.02.009. Epub 2020 Mar 3. Way KL J Sport Health Sci 2021 24-05-2020 PMC8343005.
56. 27782779 Effects of Endurance Training on the Skeletal Muscle Nitric Oxide Metabolism in Insulin-Independent Type 2 Diabetic Men-A Pilot Study Brinkmann C, Schulte-Körne B, Grau M, Obels S, Kemmerling R, Schiffer T, Bloch W, Brixius K. Metab Syndr Relat Disord. 2017 Feb;15(1):52-58. doi: 10.1089/met.2016.0092. Epub 2016 Oct 26. Brinkmann C Metab Syndr Relat Disord 2017 27-10-2016.
57. 32598997 The effect of low-volume high-intensity interval training on cardiovascular health outcomes in type 2 diabetes: A randomized controlled trial Way KL, Sabag A, Sultana RN, Baker MK, Keating SE, Lanting S, Gerofi J, Chuter VH, Caterson ID, Twigg SM, Johnson NA. Int J Cardiol. 2020 Dec 1; 320:148-154. doi: 10.1016/j.ijcard.2020.06.019. Epub 2020 Jun 26. Way KL Int J Cardiol 2020 30-06-2020
58. 37057183 The effects of pedometer-based exercise on central and peripheral vascular functions among young sedentary men with CVD risk factors Omar N, Yeoh BS, Chellappan K, Chui SZ, Salamt N, Aminuddin A. Front Physiol. 2023 Mar 28; 14:1062751. doi: 10.3389/fphys.2023.1062751. eCollection 2023. Omar N Front Physiol 2023 14-04-2023 PMC10088098.
59. 28708930 Effects of high-intensity aerobic interval training on cardiovascular disease risk in testicular cancer survivors: A phase 2 randomized controlled trial Adams SC, DeLorey DS, Davenport MH, Stickland MK, Fairey AS, North S, Szczotka A, Courneya KS. Cancer. 2017 Oct 15;123(20):4057-4065. doi: 10.1002/cncr.30859. Epub 2017 Jul 14. Adams SC Cancer 2017 15-07-2017.
60. 18673303 Aerobic interval training reduces cardiovascular risk factors more than a multitreatment approach in overweight adolescents Tjønna AE, Stølen TO, Bye A, Volden M, Slørdahl SA, Odegård R, Skogvoll E, Wisløff U. Clin Sci (Lond). 2009 Feb;116(4):317-26. doi: 10.1042/CS20080249. Tjønna AE Clin Sci (Lond) 2009 05-08-2008.
61. 34111245 Concomitant Peripheral Neuropathy and Type 2 Diabetes Impairs Postexercise Cutaneous Perfusion and Flowmotion Reynès C, Beaume JB, Latil-Plat F, Ennaifer H, Rocher L, Antoine-Jonville S, Benamo E, Knapp Y, Vinet A. J Clin Endocrinol Metab. 2021 Sep 27;106(10): e3979-e3989. doi: 10.1210/clinem/dgab414. Reynès CJ Clin Endocrinol Metab 2021 10-06-2021.
62. 21977456 Effects of Aerobic Exercise vs. Resistance Training on Endothelial Function in Women with Type 2 Diabetes Mellitus Kwon HR, Min KW, Ahn HJ, Seok HG, Lee JH, Park GS, Han KA. Diabetes Metab J. 2011 Aug;35(4):364-73. doi: 10.4093/dmj.2011.35.4.364. Epub 2011 Aug 31. Kwon HR Diabetes Metab J 2011 07-10-2011 PMC3178697.
63. 30137674 Influence of body composition and physical fitness on arterial stiffness after marathon running Deiseroth A, Nussbaumer M, Drexel V, Hertel G, Schmidt-Trucksäss A, Vlachopoulos C, Halle M, Hanssen H. Scand J Med Sci Sports. 2018 Dec;28(12):2651-2658. doi: 10.1111/sms.13283. Epub 2018 Sep 10. Deiseroth A Scand J Med Sci Sports 2018 24-08-2018.
64. 19609554 Short-term exercise training improves aerobic capacity with no change in arterial function in obesity Baynard T, Carhart RL Jr, Weinstock RS, Ploutz-Snyder LL, Kanaley JA. Eur J Appl Physiol. 2009 Oct;107(3):299-308. doi: 10.1007/s00421-009-1126-2. Epub 2009 Jul 16. Baynard T Eur J Appl Physiol 2009 18-07-2009.
65. 33905304 Effects of chronic dietary grape seed extract supplementation on aortic stiffness and hemodynamic responses in obese/overweight males during submaximal exercise Dillon K, Shariffi B, Gillum T, Boyer W, Sullivan S, Kim JK. Eur J Sport Sci. 2022 Jul;22(7):1057-1064. doi: 10.1080/17461391.2021.1923815. Epub 2021 May 20. Dillon K Eur J Sport Sci 2022 27-04-2021.
66. 28302517 Exercise training rescues high fat diet-induced neuronal nitric oxide synthase expression in the hippocampus and cerebral cortex of mice Tomiga Y, Yoshimura S, Ito A, Nakashima S, Kawanaka K, Uehara Y, Tanaka H, Higaki Y. Nitric Oxide. 2017 Jun 1; 66:71-77. doi: 10.1016/j.niox.2017.03.002. Epub 2017 Mar 14. Tomiga Y Nitric Oxide 2017 18-03-2017.
67. 32467641 The impact of high-intensity interval training (HIIT) and moderate-intensity continuous training (MICT) on arterial stiffness and blood pressure in young obese women: a randomized controlled trial de Oliveira GH, Boutouyrie P, Simões CF, Locatelli JC, Mendes VHS, Reck HB, Costa CE, Okawa RTP, Lopes WA. Hypertens Res. 2020 Nov;43(11):1315-1318. doi: 10.1038/s41440-020-0477-2. Epub 2020 May 29. de Oliveira GH Hypertens Res 2020 30-05-2020.
68. 37313274 Effects of a Mobile-Health Exercise Intervention on Body Composition, Vascular Function, and Autonomic Nervous System Function in Obese Women: A Randomized Controlled Trial Choi JH, Kim SW, Seo J, Sun Y, Jung WS, Park HY, Kim J, Lim K. J Multidiscip Healthc. 2023 Jun 8; 16:1601-1615. doi: 10.2147/JMDH.S406905. eCollection 2023.Choi JH J Multidiscip Healthc 2023 14-06-2023 PMC10259526.
69. 15580192 Inflammation, insulin, and endothelial function in overweight children and adolescents: the role of exercise Kelly AS, Wetzsteon RJ, Kaiser DR, Steinberger J, Bank AJ, Dengel DR. J Pediatr. 2004 Dec;145(6):731-6. doi: 10.1016/j.jpeds.2004.08.004. Kelly AS J Pediatr 2004 08-12-2004.
70. 30537411 Postprandial augmentation index is reduced in adults with prediabetes following continuous and interval exercise training Eichner NZM, Gaitán JM, Gilbertson NM, Khurshid M, Weltman A, Malin SK. Exp Physiol. 2019 Feb;104(2):264-271. doi: 10.1113/EP087305. Epub 2018 Dec 27. Eichner NZM Exp Physiol 2019 12-12-2018.
71. 34816612 Effects of indulgent food snacking, with and without exercise training, on body weight, fat mass, and cardiometabolic risk markers in overweight and obese men Tucker WJ, Jarrett CL, D'Lugos AC, Angadi SS, Gaesser GA. Physiol Rep. 2021 Nov;9(22): e15118. doi: 10.14814/phy2.15118. Tucker WJ Physiol Rep 2021 24-11-2021 PMC8611507.
72. 34504188 Pleiotropic effects between cardiovascular disease risk factors and measures of cognitive and physical function in long-lived adults Yudkovicz JJ, Minster RL, Barinas-Mitchell E, Christensen K, Feitosa M, Barker MS, Newman AB, Kuipers AL. Sci Rep. 2021 Sep 9;11(1):17980. doi: 10.1038/s41598-021-97298-0. Yudkovicz JJ Sci Rep 2021 10-09-2021 PMC8429644.
73. 24534270 Accumulated brisk walking reduces arterial stiffness in overweight adults: evidence from a randomized control trial Kearney TM, Murphy MH, Davison GW, O'Kane MJ, Gallagher AM. J Am Soc Hypertens. 2014 Feb;8(2):117-26. doi: 10.1016/j.jash.2013.10.001. Epub 2013 Oct 6. Kearney TM J Am Soc Hypertens 2014 19-02-2014.
74. 29892497 Fatness and Fluctuating Body Weight: Effect on Central Vasculature Zeigler ZS, Birchfield N, Moreno K, James D, Swan P. Biores Open Access. 2018 Jun 1;7(1):90-100. doi: 10.1089/biores.2017.0044. eCollection 2018. Zeigler ZS Biores Open Access 2018 13-06-2018 PMC5994146.
75. 33953580 The Associations of Perceived and Oxidative Stress with Hypertension in a Cohort of Police Officers Janczura M, Rosa R, Dropinski J, Gielicz A, Stanisz A, Kotula-Horowitz K, Domagala T. Diabetes Metab Syndr Obes. 2021 Apr 23; 14:1783-1797. doi: 10.2147/DMSO.S298596. eCollection 2021. Janczura M Diabetes Metab Syndr Obes 2021 06-05-2021 PMC8090790.
76. 34912517 The Effect of All Extremity High Intensity Interval Training on Athero-Protective Factors and Endothelial Function in Overweight and Obese Women Hovsepian V, Marandi SM, Esfarjani F, Zavar R, Sadeghi M. Int J Prev Med. 2021 Oct 26; 12:141. doi: 10.4103/ijpvm.IJPVM_248_19. eCollection 2021. Hovsepian V Int J Prev Med 2021 16-12-2021 PMC8631139.
77. 33921520 Endurance Training Depletes Antioxidant System but Does Not Affect Endothelial Functions in Women with Abdominal Obesity: A Randomized Trial with a Comparison to Endurance-Strength Training Jamka M, Bogdański P, Krzyżanowska-Jankowska P, Miśkiewicz-Chotnicka A, Karolkiewicz J, Duś-Żuchowska M, Mądry R, Lisowska A, Gotz-Więckowska A, Iskakova S, Walkowiak J, Mądry E. J Clin Med. 2021 Apr 12;10(8):1639. doi: 10.3390/jcm10081639. Jamka M J Clin Med 2021 30-04-2021 PMC8068807.
78. 37611320 Effects of crossover point exercise and high-intensity interval training on vascular health in young overweight females Yang Y, Zhang P, Zhu X. Appl Physiol Nutr Metab. 2024 Jan 1;49(1):77-86. doi: 10.1139/apnm-2023-0054. Epub 2023 Aug 23. Yang Y Appl Physiol Nutr Metab 2024 23-08-2023.
79. 26953821 Systolic blood pressure response after high-intensity interval exercise is independently related to decreased small arterial elasticity in normotensive African American women Carter SJ, Goldsby TU, Fisher G, Plaisance EP, Gower BA, Glasser SP, Hunter GR. Appl Physiol Nutr Metab. 2016 May;41(5):484-90. doi: 10.1139/apnm-2015-0512. Epub 2016 Jan 7. Carter SJ Appl Physiol Nutr Metab 2016 09-03-2016 PMC4854761NIHMS771876.
80. 28835674 Soy Improves Cardiometabolic Health and Cecal Microbiota in Female Low-Fit Rats Cross TL, Zidon TM, Welly RJ, Park YM, Britton SL, Koch LG, Rottinghaus GE, de Godoy MRC, Padilla J, Swanson KS, Vieira-Potter VJ. Sci Rep. 2017 Aug 23;7(1):9261. doi: 10.1038/s41598-017-08965-0. Cross TL Sci Rep 2017 25-08-2017 PMC5569109.
81. 22809789 Expression of cardiac GATA4 and downstream genes after exercise training in the db/db mouse Broderick TL, Parrott CR, Wang D, Jankowski M, Gutkowska J. Pathophysiology. 2012 Jun;19(3):193-203. doi: 10.1016/j.pathophys.2012.06.001. Epub 2012 Jul 17. Broderick TL Pathophysiology 2012 20-07-2012.
82. 33873267 Impact of prolonged sitting and physical activity breaks on cognitive performance, perceivable benefits, and cardiometabolic health in overweight/obese adults: The role of meal composition Wanders L, Cuijpers I, Kessels RPC, van de Rest O, Hopman MTE, Thijssen DHJ. Clin Nutr. 2021 Apr;40(4):2259-2269. doi: 10.1016/j.clnu.2020.10.006. Epub 2020 Oct 9. Wanders L Clin Nutr 2021 20-04-2021.
83. 27342073 High Intensity Interval- vs Resistance or Combined- Training for Improving Cardiometabolic Health in Overweight Adults (Cardiometabolic HIIT-RT Study): study protocol for a randomized controlled trial Ramírez-Vélez R, Hernandez A, Castro K, Tordecilla-Sanders A, González-Ruíz K, Correa-Bautista JE, Izquierdo M, García-Hermoso A. Trials. 2016 Jun 24;17(1):298. doi: 10.1186/s13063-016-1422-1. Ramírez-Vélez R Trials 2016 26-06-2016 PMC4919882.
84. 27044585 Effects of exercise intensity and nutrition advice on myocardial function in obese children and adolescents: a multicentre randomized controlled trial study protocol Dias KA, Coombes JS, Green DJ, Gomersall SR, Keating SE, Tjonna AE, Hollekim-Strand SM, Hosseini MS, Ro TB, Haram M, Huuse EM, Davies PS, Cain PA, Leong GM, Ingul CB. BMJ Open. 2016 Apr 4;6(4): e010929. doi: 10.1136/bmjopen-2015-010929. Dias KA BMJ Open 2016 06-04-2016 PMC4823457.
85. 28138261 The effects of exercise training and caloric restriction on the cardiac oxytocin natriuretic peptide system in the diabetic mouse Broderick TL, Jankowski M, Gutkowska J. Diabetes Metab Syndr Obes. 2017 Jan 11; 10:27-36. doi: 10.2147/DMSO.S115453. eCollection 2017. Broderick TL Diabetes Metab Syndr Obes 2017 01-02-2017 PMC5238809.
86. 27088872 Obesity and the Role of Short Duration Submaximal Work on Cardiovascular and Cerebral Hemodynamics Cavuoto LA, Maikala RV. PLoS One. 2016 Apr 18;11(4):e0153826. doi: 10.1371/journal.pone.0153826. eCollection 2016. Cavuoto LA PLoS One 2016 19-04-2016 PMC4835079.
87. 31676260 Effects of high-intensity interval training on endothelial function, lipid profile, body composition and physical fitness in normal-weight and overweight-obese adolescents: A clinical trialda Silva MR, Waclawovsky G, Perin L, Camboim I, Eibel B, Lehnen AM. Physiol Behav. 2020 Jan 1; 213:112728. doi: 10.1016/j.physbeh.2019.112728. Epub 2019 Oct 30. da Silva MR Physiol Behav 2020 03-11-2019.
88. 29872225 Reducing sitting time versus adding exercise: differential effects on biomarkers of endothelial dysfunction and metabolic risk Duvivier BMFM, Bolijn JE, Koster A, Schalkwijk CG, Savelberg HHCM, Schaper NC. Sci Rep. 2018 Jun 5;8(1):8657. doi: 10.1038/s41598-018-26616-w. Duvivier BMFM Sci Rep 2018 07-06-2018 PMC5988819.
89. 27250851 Exercise prior to assisted fertilization in overweight and obese women (FertilEX): study protocol for a randomized controlled trial Lundgren KM, Romundstad LB, von Düring V, Mørkved S, Kjøtrød S, Moholdt T. Trials. 2016 Jun 1;17(1):268. doi: 10.1186/s13063-016-1398-x. Lundgren KM Trials 2016 03-06-2016 PMC4888643.
90. 32975908 Exercise during pregnancy mitigates the adverse effects of maternal obesity on adult male offspring vascular function and alters one-carbon metabolism Boonpattrawong NP, Golbidi S, Tai DC, Aleliunas RE, Bernatchez P, Miller JW, Laher I, Devlin AM. Physiol Rep. 2020 Sep;8(18):e14582. doi: 10.14814/phy2.14582. Boonpattrawong NP Physiol Rep 2020 25-09-2020 PMC7518297.
91. 29515458 Exercise, Arterial Crosstalk-Modulation, and Inflammation in an Aging Population: The ExAMIN AGE Study Streese L, Deiseroth A, Schäfer J, Schmidt-Trucksäss A, Hanssen H. Front Physiol. 2018 Feb 21; 9:116. doi: 10.3389/fphys.2018.00116. eCollection 2018. Streese L Front Physiol 2018 09-03-2018 PMC5826378.
92. 21737826 Seven days of aerobic exercise training improves conduit artery blood flow following glucose ingestion in patients with type 2 diabetes Mikus CR, Fairfax ST, Libla JL, Boyle LJ, Vianna LC, Oberlin DJ, Uptergrove GM, Deo SH, Kim A, Kanaley JA, Fadel PJ, Thyfault JP. J Appl Physiol (1985). 2011 Sep;111(3):657-64. doi: 10.1152/japplphysiol.00489.2011. Epub 2011 Jul 7. Mikus CR J Appl Physiol (1985) 2011 09-07-2011 PMC3174788.
93. 35081314 ARTE index revisited: linking biomarkers of cardiometabolic health with free-living physical activity in postmenopausal women Carter SJ, Baranauskas MN, Singh H, Martins C, Hunter GR. Am J Physiol Regul Integr Comp Physiol. 2022 Apr 1;322(4):R292-R298. doi: 10.1152/ajpregu.00075.2021. Epub 2022 Jan 26. Carter SJ Am J Physiol Regul Integr Comp Physiol 2022 26-01-2022 PMC8917908 10.1152/ajpregu.00075.2021.
94. 25029200 Lifestyle modification decreases arterial stiffness in overweight and obese men: dietary modification vs. exercise training Maeda S, Zempo-Miyaki A, Sasai H, Tsujimoto T, So R, Tanaka K. Int J Sport Nutr Exerc Metab. 2015 Feb;25(1):69-77. doi: 10.1123/ijsnem.2013-0107. Epub 2014 Jul 14. Maeda S Int J Sport Nutr Exerc Metab 2015 17-07-2014.
95. 25580979 [High versus moderate intense running exercise - effects on cardiometabolic risk factors in untrained males] Kemmler W, Lell M, Scharf M, Fraunberger L, von Stengel S. Dtsch Med Wochenschr. 2015 Jan;140(1): e7-e13. doi: 10.1055/s-0040-100423. Epub 2015 Jan 12. Kemmler W Dtsch Med Wochenschr 2015 13-01-2015.
96. 30758976 A free-choice high-fat, high-sucrose diet induces hyperphagia, obesity, and cardiovascular dysfunction in female cycling and pregnant rats Ahmed H, Hannan JL, Apolzan JW, Osikoya O, Cushen SC, Romero SA, Goulopoulou S. Am J Physiol Regul Integr Comp Physiol. 2019 May 1;316(5): R472-R485. doi: 10.1152/ajpregu.00391.2018. Epub 2019 Feb 13. Ahmed H Am J Physiol Regul Integr Comp Physiol 2019 14-02-2019 PMC6589604.
97. 22976739 Effects of 12 weeks of aerobic exercise on body composition and vascular compliance in obese boys Song JK, Stebbins CL, Kim TK, Kim HB, Kang HJ, Chai JH. J Sports Med Phys Fitness. 2012 Oct;52(5):522-9. Song JK J Sports Med Phys Fitness 2012 15-09-2012.
98. 32815632 Physical activity is associated with lower arterial stiffness in normal-weight postmenopausal women Stamatelopoulos K, Tsoltos N, Armeni E, Paschou SA, Augoulea A, Kaparos G, Rizos D, Karagouni I, Delialis D, Ioannou S, Apostolakis M, Makrakis E, Lambrinoudaki I. J Clin Hypertens (Greenwich). 2020 Sep;22(9):1682-1690. doi: 10.1111/jch.13954. Epub 2020 Aug 20. Stamatelopoulos K J Clin Hypertens (Greenwich) 2020 21-08-2020 PMC8029682.
99. 26172834 Voluntary Exercise Can Ameliorate Insulin Resistance by Reducing iNOS-Mediated S-Nitrosylation of Akt in the Liver in Obese Rats Tsuzuki T, Shinozaki S, Nakamoto H, Kaneki M, Goto S, Shimokado K, Kobayashi H, Naito H. PLoS One. 2015 Jul 14;10(7): e0132029. doi: 10.1371/journal.pone.0132029. eCollection 2015.Tsuzuki T PLoS One 2015 15-07-2015 PMC4501.
100. 32577192 The Impact of High-Intensity Interval Training Versus Moderate-Intensity Continuous Training on Carotid Intima-Media Thickness and Ankle-Brachial Index in Middle-Aged Women Farahati S, Hosseini SRA, Moazzami M, Daloee MH, Daloee SH. Int J Prev Med. 2020 Jun 3; 11:62. doi: 10.4103/ijpvm.IJPVM_524_18. eCollection 2020. Farahati S Int J Prev Med 2020 25-06-2020 PMC7297415.
101. 32187150 The Effect of 12 Weeks of Different Exercise Training Modalities or Nutritional Guidance on Cardiometabolic Risk Factors, Vascular Parameters, and Physical Fitness in Overweight Adults: Cardiometabolic High-Intensity Interval Training-Resistance Training Randomized Controlled Study Ramírez-Vélez R, Castro-Astudillo K, Correa-Bautista JE, González-Ruíz K, Izquierdo M, García-Hermoso A, Álvarez C, Ramírez-Campillo R, Correa-Rodríguez M. J Strength Cond Res. 2020 Aug;34(8):2178-2188. doi: 10.1519/JSC.0000000000003533. Ramírez-Vélez RJ Strength Cond Res 2020 19-03-2020.
102. 23346301 Aged garlic extract enhances exercise-mediated improvement of metabolic parameters in high fat diet-induced obese rats Seo DY, Lee S, Figueroa A, Kwak YS, Kim N, Rhee BD, Ko KS, Bang HS, Baek YH, Han J. Nutr Res Pract. 2012 Dec;6(6):513-9. doi: 10.4162/nrp.2012.6.6.513. Epub 2012 Dec 31. Seo DY Nutr Res Pract 2012 25-01-2013 PMC3542441.
103. 29932503 Physical activity protects NLRP3 inflammasome-associated coronary vascular dysfunction in obese mice Lee J, Lee Y, LaVoy EC, Umetani M, Hong J, Park Y. Physiol Rep. 2018 Jun;6(12): e13738. doi: 10.14814/phy2.13738. Lee J Physiol Rep 2018 23-06-2018 PMC6014.
104. 29589111 Effects of resistance training on MRI-derived epicardial fat volume and arterial stiffness in women with obesity: a randomized pilot study Fernandez-del-Valle M, Gonzales JU, Kloiber S, Mitra S, Klingensmith J, Larumbe-Zabala E. Eur J Appl Physiol. 2018 Jun;118(6):1231-1240. doi: 10.1007/s00421-018-3852-9. Epub 2018 Mar 27. Fernandez-del-Valle M Eur J Appl Physiol 2018 29-03-2018.
105. 16783277 [Physical activity in hypertension management] Chanudet X, Lambert de Cremeur G, Bonnevie L.Presse Med. 2006 Jun;35(6 Pt 2):1081-7. doi: 10.1016/s0755-4982(06)74752-2. Chanudet X Presse Med 2006 20-06-2006.
106. 32923415 Gestational Diabetes: Physical Activity Before Pregnancy and Its Influence on the Cardiovascular System Sitzberger C, Oberhoffer-Fritz R, Meyle K, Wagner M, Lienert N, Graupner O, Ensenauer R, Lobmaier SM, Wacker-Gußmann A. Front Pediatr. 2020 Aug 14; 8:465. doi: 10.3389/fped.2020.00465. eCollection 2020. Sitzberger C Front Pediatr 2020 14-09-2020 PMC7456967.
107. 23722526 Improvement of exercise capacity and peripheral metaboreflex after bariatric surgery da Silva RP, Martinez D, Faria CC, de Carli LA, de Souza WI, Meinhardt NG, Souto KE, Trindade MR, Ribeiro JP. Obes Surg. 2013 Nov;23(11):1835-41. doi: 10.1007/s11695-013-0988-x. da Silva RP Obes Surg 2013 01-06-2013.
108. 29619067A Hop Extract Lifenol® Improves Postmenopausal Overweight, Osteoporosis, and Hot Flash in Ovariectomized Rats Ban YH, Yon JM, Cha Y, Choi J, An ES, Guo H, Seo DW, Kim TS, Lee SP, Kim JC, Choi EK, Kim YB. Evid Based Complement Alternat Med. 2018 Feb 12; 2018:2929107. doi: 10.1155/2018/2929107. eCollection 2018. Ban YH Evid Based Complement Alternat Med 2018 06-04-2018 PMC5829324.
109. 30803498 The effect of high Intensity interval training versus moderate intensity continuous training on arterial stiffness and 24h blood pressure responses: A systematic review and meta-analysis Way KL, Sultana RN, Sabag A, Baker MK, Johnson NA. J Sci Med Sport. 2019 Apr;22(4):385-391. doi: 10.1016/j.jsams.2018.09.228. Epub 2018 Sep 22. Way KL J Sci Med Sport 2019 27-02-2019.
110. 32595244 Safety and effects of crataegus extract ws 1442 and nordic walking on lipid profile and endothelial function: a randomized, partially blinded pilot study in overweight volunteers Niederseer D, Ledl-Kurkowski E, Kvita K, Funk P, Niebauer J. Acta Clin Croat. 2019 Dec;58(4):604-614. doi: 10.20471/acc.2019.58.04.06. Niederseer D Acta Clin Croat 2019 30-06-2020 PMC7314302.
111. 30584131 Acute L-Arginine supplementation does not affect red blood cell aggregation and deformability during high intensity interval exercise in healthy men Ahmadizad S, Daraei A, Bassami M, Rahmani H. Clin Hemorheol Microcirc. 2019;71(2):215-223. doi: 10.3233/CH-189413. Ahmadizad S Clin Hemorheol Microcirc 2019 26-12-2018.
112. 27807171 Effects of Long-Term Exercise on Age-Related Hearing Loss in Mice Han C, Ding D, Lopez MC, Manohar S, Zhang Y, Kim MJ, Park HJ, White K, Kim YH, Linser P, Tanokura M, Leeuwenburgh C, Baker HV, Salvi RJ, Someya S. J Neurosci. 2016 Nov 2;36(44):11308-11319. doi: 10.1523/JNEUROSCI.2493-16.2016. Han CJ Neurosci 2016 04-11-2016 PMC5148246.
113. 25645978 Sprint interval and moderate-intensity continuous training have equal benefits on aerobic capacity, insulin sensitivity, muscle capillarisation and endothelial eNOS/NAD(P)Hoxidase protein ratio in obese men Cocks M, Shaw CS, Shepherd SO, Fisher JP, Ranasinghe A, Barker TA, Wagenmakers AJ. J Physiol. 2016 Apr 15;594(8):2307-21. doi: 10.1113/jphysiol.2014.285254. Epub 2015 Feb 24. Cocks MJ Physiol 2016 04-02-2015 PMC4933110.
114. 27255523 Effects of high-intensity interval training and moderate-intensity continuous training on endothelial function and cardiometabolic risk markers in obese adults Sawyer BJ, Tucker WJ, Bhammar DM, Ryder JR, Sweazea KL, Gaesser GA. J Appl Physiol (1985). 2016 Jul 1;121(1):279-88. doi: 10.1152/japplphysiol.00024.2016. Epub 2016 Jun 2. Sawyer BJ; J Appl Physiol (1985) 2016 04-06-2016 PMC4967258.
115. 12793971 Physical activity and the prevention of cardiovascular disease Bassuk SS, Manson JE. Curr Atheroscler Rep. 2003 Jul;5(4):299-307. doi: 10.1007/s11883-003-0053-7. Bassuk SS Curr Atheroscler Rep 2003 10-06-2003.
116. 30193792 Endothelial function is disturbed in a hypertensive diabetic animal model of HFpEF: Moderate continuous vs. high intensity interval training Schmederer Z, Rolim N, Bowen TS, Linke A, Wisloff U, Adams V; OptimEx study group. Int J Cardiol. 2018 Dec 15; 273:147-154. doi: 10.1016/j.ijcard.2018.08.087. Epub 2018 Aug 30.Schmederer Z Int J Cardiol 2018 09-09-2018.
117. 32605284 Acute Effects of Beetroot Juice Supplements on Resistance Training: A Randomized Double-Blind Crossover Ranchal-Sanchez A, Diaz-Bernier VM, De La Florida-Villagran CA, Llorente-Cantarero FJ, Campos-Perez J, Jurado-Castro JM. Nutrients. 2020 Jun 28;12(7):1912. doi: 10.3390/nu12071912. Ranchal-Sanchez A Nutrients 2020 02-07-2020 PMC7401280.
118. 27247333 Restoration of Mitochondrial Cardiolipin Attenuates Cardiac Damage in Swine Renovascular Hypertension Eirin A, Ebrahimi B, Kwon SH, Fiala JA, Williams BJ, Woollard JR, He Q, Gupta RC, Sabbah HN, Prakash YS, Textor SC, Lerman A, Lerman LO. J Am Heart Assoc. 2016 May 31;5(6):e003118. doi: 10.1161/JAHA.115.003118. Eirin A J Am Heart Assoc 2016 02-06-2016 PMC4937260.
119. 29693429 Ergogenic effects of beetroot juice supplementation during severe-intensity exercise in obese adolescents Rasica L, Porcelli S, Marzorati M, Salvadego D, Vezzoli A, Agosti F, De Col A, Tringali G, Jones AM, Sartorio A, Grassi B. Am J Physiol Regul Integr Comp Physiol. 2018 Sep 1;315(3):R453-R460. doi: 10.1152/ajpregu.00017.2018. Epub 2018 Apr 25. Rasica L Am J Physiol Regul Integr Comp Physiol 2018 26-04-2018.
120. 25936306 Impact of high-fat diet and voluntary running on body weight and endothelial function in LDL receptor knockout mice Langbein H, Hofmann A, Brunssen C, Goettsch W, Morawietz H. Atheroscler Suppl. 2015 May;18:59-66. doi: 10.1016/j.atherosclerosissup.2015.02.010. Langbein H Atheroscler Suppl 2015 05-05-2015.
121. 10023732 Changes in skeletal muscle vascular resistance with weight gain: associations with insulin and sympathetic activity Borne AT, Truett AA, Monteiro MP, Volaufova J, West DB. Obes Res. 1999 Jan;7(1):68-75. doi: 10.1002/j.1550-8528.1999.tb00392.x. Borne AT Obes Res 1999 19-02-1999.
122. 6756274 Obesity, diabetes mellitus and physical activity–metabolic responses to physical training in adipose and muscle tissues Sullivan L. Ann Clin Res. 1982;14 Suppl 34:51-62. Sullivan L Ann Clin Res 198 201-01-1982.
123. 26379244 Chronic Running Exercise Alleviates Early Progression of Nephropathy with Upregulation of Nitric Oxide Synthases and Suppression of Glycation in Zucker Diabetic Rats Ito D, Cao P, Kakihana T, Sato E, Suda C, Muroya Y, Ogawa Y, Hu G, Ishii T, Ito O, Kohzuki M, Kiyomoto H. PLoS One. 2015 Sep 17;10(9):e0138037. doi: 10.1371/journal.pone.0138037. eCollection 2015. Ito D PLoS One 2015 18-09-2015 PMC4574951.
124. 22552687 The effect of diet and exercise on markers of endothelial function in overweight and obese women with polycystic ovary syndrome Thomson RL, Brinkworth GD, Noakes M, Clifton PM, Norman RJ, Buckley JD. Hum Reprod. 2012 Jul;27(7):2169-76. doi: 10.1093/humrep/des138. Epub 2012 May 2. Thomson RL Hum Reprod 2012 04-05-2012.
125. 31800011 NADPH oxidase 4 mediates the protective effects of physical activity against obesity-induced vascular dysfunction Brendel H, Shahid A, Hofmann A, Mittag J, Bornstein SR, Morawietz H, Brunssen C. Cardiovasc Res. 2020 Aug 1;116(10):1767-1778. doi: 10.1093/cvr/cvz322. Brendel H Cardiovasc Res 2020 05-12-2019.
126. 15607401 Effects of prior moderate exercise on postprandial metabolism and vascular function in lean and centrally obese men Gill JM, Al-Mamari A, Ferrell WR, Cleland SJ, Packard CJ, Sattar N, Petrie JR, Caslake MJ. J Am Coll Cardiol. 2004 Dec 21;44(12):2375-82. doi: 10.1016/j.jacc.2004.09.035. Gill JM J Am Coll Cardiol 2004 21-12-2004.
127. 27286178 Repeated Warm Water Immersion Induces Similar Cerebrovascular Adaptations to 8 Weeks of Moderate-Intensity Exercise Training in Females Bailey TG, Cable NT, Miller GD, Sprung VS, Low DA, Jones H. Int J Sports Med. 2016 Sep;37(10):757-65. doi: 10.1055/s-0042-106899. Epub 2016 Jun 10. Bailey TG Int J Sports Med 2016 11-06-2016.
128. 28060442 Effects of 6 months of aerobic and resistance exercise training on carotid artery intima media thickness in overweight and obese older women Park J, Park H. Geriatr Gerontol Int. 2017 Dec;17(12):2304-2310. doi: 10.1111/ggi.12972. Epub 2017 Jan 6. Park J Geriatr Gerontol Int 2017 07-01-2017.
129. 34247226 Acute effects of high-intensity interval training and moderate-intensity continuous training on arterial stiffness in young obese women Hortmann K, Boutouyrie P, Locatelli JC, de Oliveira GH, Simões CF, de Souza Mendes VH, Reck HB, Okawa RTP, Lopes WA. Eur J Prev Cardiol. 2021 Jul 10;28(7):e7-e10. doi: 10.1177/2047487320909302. Hortmann K Eur J Prev Cardiol 2021 11-07-2021.
130. 24381004 Exercise training boosts eNOS-dependent mitochondrial biogenesis in mouse heart: role in adaptation of glucose metabolism Vettor R, Valerio A, Ragni M, Trevellin E, Granzotto M, Olivieri M, Tedesco L, Ruocco C, Fossati A, Fabris R, Serra R, Carruba MO, Nisoli E. Am J Physiol Endocrinol Metab. 2014 Mar 1;306(5): E519-28. doi: 10.1152/ajpendo.00617.2013. Epub 2013 Dec 31. Vettor R Am J Physiol Endocrinol Metab 2014 02-01-2014.
131. 28716522 Exercise individualized by TRIMPi method reduces arterial stiffness in early onset type 2 diabetic patients: A randomized controlled trial with aerobic interval training Bellia A, Iellamo F, De Carli E, Andreadi A, Padua E, Lombardo M, Annino G, Campoli F, Tartaglione S, D'Ottavio S, Della-Morte D, Lauro D. Int J Cardiol. 2017 Dec 1; 248:314-319. doi: 10.1016/j.ijcard.2017.06.065. Epub 2017 Jun 17. Bellia A Int J Cardiol 2017 19-07-2017.
132. 25802037 A randomized trial comparing the effect of exercise training and weight loss on microvascular function in coronary artery disease Olsen RH, Pedersen LR, Jürs A, Snoer M, Haugaard SB, Prescott E. Int J Cardiol. 2015 Apr 15; 185:229-35. doi: 10.1016/j.ijcard.2015.03.118. Epub 2015 Mar 11. Olsen RH Int J Cardiol 2015 25-03-2015.
133. 17088459 Impaired chronotropic and vasodilator reserves limit exercise capacity in patients with heart failure and a preserved ejection fraction Borlaug BA, Melenovsky V, Russell SD, Kessler K, Pacak K, Becker LC, Kass DA. Circulation. 2006 Nov 14;114(20):2138-47. doi: 10.1161/CIRCULATIONAHA.106.632745. Epub 2006 Nov 6. Borlaug BA Circulation 2006 08-11-2006.
134. 26433119 Combined omega-3 fatty acids, aerobic exercise and cognitive stimulation prevents decline in gray matter volume of the frontal, parietal and cingulate cortex in patients with mild cognitive impairment Köbe T, Witte AV, Schnelle A, Lesemann A, Fabian S, Tesky VA, Pantel J, Flöel A. Neuroimage. 2016 May 1; 131:226-38. doi: 10.1016/j.neuroimage.2015.09.050. Epub 2015 Oct 1. Köbe T Neuroimage 2016 04-10-2015.
135. 20877288 Vascular reactivity at rest and during exercise in middle-aged obese men: effects of short-term, low-intensity, exercise training Vinet A, Karpoff L, Walther G, Startun A, Obert P, Goret L, Dauzat M, Perez-Martin A. Int J Obes (Lond). 2011 Jun;35(6):820-8. doi: 10.1038/ijo.2010.206. Epub 2010 Sep 28. Vinet A Int J Obes (Lond) 2011 30-09-2010.
136. 36807940 Impact of body mass index on markers of vascular health in normotensive women with history of pre-eclampsia Heidema WH, Van Drongelen J, Spaanderman MEA, Scholten RR. Ultrasound Obstet Gynecol. 2023 Jul;62(1):122-129. doi: 10.1002/uog.26182. Heidema WH Ultrasound Obstet Gynecol 2023 22-02-2023.
137. 30379310 Survey on Physical Fitness and Cardiovascular Function of the City Elderly in Different Regular Physical Activities in China Chen L, Wang S, Xu JC. J Nutr Health Aging. 2018;22(9):1107-1111. doi: 10.1007/s12603-018-1070-0. Chen LJ Nutr Health Aging 2018 01-11-2018.
138. 19596737 Playing active video games increases energy expenditure in children Graf DL, Pratt LV, Hester CN, Short KR. Pediatrics. 2009 Aug;124(2):534-40. doi: 10.1542/peds.2008-2851. Epub 2009 Jul 13. Graf DL Pediatrics 2009 15-07-2009 PMC8329994NIHMS1727662.
139. 25785928 Adipose Tissue Insulin Action and IL-6 Signaling after Exercise in Obese MiceMacpherson RE, Huber JS, Frendo-Cumbo S, Simpson JA, Wright DC. Med Sci Sports Exerc. 2015 Oct;47(10):2034-42. doi: 10.1249/MSS.0000000000000660. Macpherson RE Med Sci Sports Exerc 2015 19-03-2015.
140. 25710559 Differential impact of acute high-intensity exercise on circulating endothelial microparticles and insulin resistance between overweight/obese males and females Durrer C, Robinson E, Wan Z, Martinez N, Hummel ML, Jenkins NT, Kilpatrick MW, Little JP. PLoS One. 2015 Feb 24;10(2): e0115860. doi: 10.1371/journal.pone.0115860. eCollection 2015. Durrer C PLoS One 2015 25-02-2015 PMC4339732 10.1371/journal.pone.0115860.
141. 23954368 Aging compounds western diet-associated large artery endothelial dysfunction in mice: prevention by voluntary aerobic exercise Lesniewski LA, Zigler ML, Durrant JR, Nowlan MJ, Folian BJ, Donato AJ, Seals DR. Exp Gerontol. 2013 Nov;48(11):1218-25. doi: 10.1016/j.exger.2013.08.001. Epub 2013 Aug 13. Lesniewski LA Exp Gerontol 2013 20-08-2013 PMC3840721NIHMS515723.
142. 31218680 Home-hit improves muscle capillarisation and eNOS/NAD(P)Hoxidase protein ratio in obese individuals with elevated cardiovascular disease risk Scott SN, Shepherd SO, Hopkins N, Dawson EA, Strauss JA, Wright DJ, Cooper RG, Kumar P, Wagenmakers AJM, Cocks M. J Physiol. 2019 Aug;597(16):4203-4225. doi: 10.1113/JP278062. Epub 2019 Jul 15. Scott SN J Physiol 2019 21-06-2019.
143. 28930627 Fitness, body composition and vascular health in adolescent and young adult survivors of pediatric brain cancer and cranial radiotherapy Long TM, Rath SR, Maroni TD, Wallman KE, Atkinson HC, Gottardo NG, Cole CH, Choong CS, Naylor LH. Int J Adolesc Med Health. 2017 Sep 20;31(5) :/j/ijamh.2019.31.issue-5/ijamh-2017-0082/ijamh-2017-0082.xml. doi: 10.1515/ijamh-2017-0082. Long TM Int J Adolesc Med Health 2017 21-09-2017.
144. 33255278 Effects of an Indoor Cycling Program on Cardiometabolic Factors in Women with Obesity vs. Normal Body Weight Ratajczak M, Skrypnik D, Krutki P, Karolkiewicz J. Int J Environ Res Public Health. 2020 Nov 24;17(23):8718. doi: 10.3390/ijerph17238718. Ratajczak M Int J Environ Res Public Health 2020 01-12-2020 PMC7727675.
145. 25514103 Walk-run training improves the anti-inflammation properties of high-density lipoprotein in patients with metabolic syndrome Sang H, Yao S, Zhang L, Li X, Yang N, Zhao J, Zhao L, Si Y, Zhang Y, Lv X, Xue Y, Qin S. J Clin Endocrinol Metab. 2015 Mar;100(3):870-9. doi: 10.1210/jc.2014-2979. Epub 2014 Dec 16. Sang H J Clin Endocrinol Metab 2015 17-12-2014.
146. 28350823 Cluster analysis to estimate the risk of preeclampsia in the high-risk Prediction and Prevention of Preeclampsia and Intrauterine Growth Restriction (PREDO) study Villa PM, Marttinen P, Gillberg J, Lokki AI, Majander K, Ordén MR, Taipale P, Pesonen A, Räikkönen K, Hämäläinen E, Kajantie E, Laivuori H. PLoS One. 2017 Mar 28;12(3): e0174399. doi: 10.1371/journal.pone.0174399. eCollection 2017.Villa PM PLoS One 2017 29-03-2017 PMC5369775.
147. 22606068 The clinical and nonclinical values of nonexercise estimation of cardiovascular endurance in young asymptomatic individuals Alomari MA, Shqair DM, Khabour OF, Alawneh K, Nazzal MI, Keewan EF. Scientific World Journal. 2012; 2012:958752. doi: 10.1100/2012/958752. Epub 2012 Apr 19. Alomari MA Scientific World Journal 2012 19-05-2012 PMC3346685.
148. 30279586 Supplementation with a selective amino acid formula ameliorates muscular dystrophy in mdx miceBanfi S, D'Antona G, Ruocco C, Meregalli M, Belicchi M, Bella P, Erratico S, Donato E, Rossi F, Bifari F, Lonati C, Campaner S, Nisoli E, Torrente Y. Sci Rep. 2018 Oct 2;8(1):14659. doi: 10.1038/s41598-018-32613-w. Banfi S Sci Rep 2018 04-10-2018 PMC6168581.
149. 17446802 Impaired postexercise cardiovascular autonomic modulation in middle-aged women with type 2 diabetes Figueroa A, Baynard T, Fernhall B, Carhart R, Kanaley JA. Eur J Cardiovasc Prev Rehabil. 2007 Apr;14(2):237-43. doi: 10.1097/HJR.0b013e32801da10d. Figueroa A Eur J Cardiovasc Prev Rehabil 2007 21-04-2007.
150. 23761637 Exercise prevents Western diet-associated erectile dysfunction and coronary artery endothelial dysfunction: response to acute apocynin and sepiapterin treatment La Favor JD, Anderson EJ, Dawkins JT, Hickner RC, Wingard CJ. Am J Physiol Regul Integr Comp Physiol. 2013 Aug 15;305(4): R423-34. doi: 10.1152/ajpregu.00049.2013. Epub 2013 Jun 12. La Favor JD Am J Physiol Regul Integr Comp Physiol 2013 14-06-2013 PMC4839473.
151. 29190761 Acute glucoregulatory and vascular outcomes of three strategies for interrupting prolonged sitting time in postmenopausal women: A pilot, laboratory-based, randomized, controlled, 4-condition, 4-period crossover trial; Kerr J, Crist K, Vital DG, Dillon L, Aden SA, Trivedi M, Castellanos LR, Godbole S, Li H, Allison MA, Khemlina GL, Takemoto ML, Schenk S, Sallis JF, Grace M, Dunstan DW, Natarajan L, LaCroix AZ, Sears DD. PLoS One. 2017 Nov 30;12(11): e0188544. doi: 10.1371/journal.pone.0188544. eCollection 2017. Kerr J PLoS One 2017 01-12-2017 PMC5708739.
152. 32614687 Skeletal muscle microvascular insulin resistance in type 2 diabetes is not improved by eight weeks of regular walking Park LK, Parks EJ, Pettit-Mee RJ, Woodford ML, Ghiarone T, Smith JA, Sales ARK, Martinez-Lemus LA, Manrique-Acevedo C, Padilla J. J Appl Physiol (1985). 2020 Aug 1;129(2):283-296. doi: 10.1152/japplphysiol.00174.2020. Epub 2020 Jul 2. Park LK J Appl Physiol (1985) 2020 03-07-2020 PMC7473951.
153. 20508468 Pre-exercise intake of different carbohydrates modifies ischemic reactive hyperemia after a session of anaerobic, but not after aerobic exercise Fernández JM, Da Silva-Grigoletto ME, Caballero-Villarraso J, Gómez-Puerto JR, Viana-Montaner BH, Tasset-Cuevas I, Túnez-Fiñana I, Pérez-Martínez P, López-Miranda J, Pérez-Jiménez F. J Strength Cond Res. 2010 Jun;24(6):1623-32. doi: 10.1519/JSC.0b013e3181d32ffc. Fernández JM J Strength Cond Res 2010 29-05-2010.
154. 14559057 Decreased energy levels can cause and sustain obesity Wlodek D, Gonzales M.J Theor Biol. 2003 Nov 7;225(1):33-44. doi: 10.1016/s0022-5193(03)00218-2. Wlodek D J Theor Biol 2003 16-10-2003.
155. 24577062 Reduced hepatic eNOS phosphorylation is associated with NAFLD and type 2 diabetes progression and is prevented by daily exercise in hyperphagic OLETF rats Sheldon RD, Laughlin MH, Rector RS. J Appl Physiol (1985). 2014 May 1;116(9):1156-64. doi: 10.1152/japplphysiol.01275.2013. Epub 2014 Feb 27. Sheldon RD J Appl Physiol (1985) 2014 01-03-2014 PMC4097825.
156. 23714599 Different modalities of exercise to reduce visceral fat mass and cardiovascular risk in metabolic syndrome: the RESOLVE randomized trial; Dutheil F, Lac G, Lesourd B, Chapier R, Walther G, Vinet A, Sapin V, Verney J, Ouchchane L, Duclos M, Obert P, Courteix D. Int J Cardiol. 2013 Oct 9;168(4):3634-42. doi: 10.1016/j.ijcard.2013.05.012. Epub 2013 May 25. Dutheil F Int J Cardiol 2013 30-05-2013.
157. 17656659 Habitual physical activity and vascular aging in a young to middle-age population at low cardiovascular risk Kozakova M, Palombo C, Mhamdi L, Konrad T, Nilsson P, Staehr PB, Paterni M, Balkau B; RISC Investigators. Stroke. 2007 Sep;38(9):2549-55. doi: 10.1161/STROKEAHA.107.484949. Epub 2007 Jul 26. Kozakova M Stroke 2007 28-07-2007.
158. 27890482 Dietary nitrate does not affect physical activity or outcomes in healthy older adults in a randomized, cross-over trial Siervo M, Oggioni C, Jakovljevic DG, Trenell M, Mathers JC, Houghton D, Celis-Morales C, Ashor AW, Ruddock A, Ranchordas M, Klonizakis M, Williams EA. Nutr Res. 2016 Dec;36(12):1361-1369. doi: 10.1016/j.nutres.2016.11.004. Epub 2016 Nov 14. Siervo M Nutr Res 2016 29-11-2016.
159. 11213561 [Drug treatment of erection disorders in patients with cardiovascular disease] Meuleman EJ, Kingma JH. Ned Tijdschr Geneeskd. 2001 Jan 27;145(4):167-71.Meuleman EJ Ned Tijdschr Geneeskd 2001 24-02-2001.
160. 31581818 Effects of diet combined with Nordic walking or walking program on weight loss and arterial stiffness in postmenopausal overweight and obese women: The Walking and Aging Verona pilot study Rossi AP, Muollo V, Fantin F, Masciocchi E, Urbani S, Taylor M, Caruso B, Milanese C, Capelli C, Schena F, Zamboni M. Eur J Prev Cardiol. 2020 Dec;27(19):2208-2211. doi: 10.1177/2047487319877712. Epub 2019 Oct 4. Rossi AP Eur J Prev Cardiol 2020 05-10-2019.
161. 30174691 Long-Term Dietary Nitrate Supplementation Does Not Prevent Development of the Metabolic Syndrome in Mice Fed a High-Fat Diet Matthews VB, Hollingshead R, Koch H, Croft KD, Ward NC. Int J Endocrinol. 2018 Aug 5; 2018:7969750. doi: 10.1155/2018/7969750. eCollection 2018. Matthews VB Int J Endocrinol 2018 04-09-2018 PMC6098922.
162. 33746114 Improvement after bariatric surgery in chronic thromboembolic pulmonary hypertension: a novel treatment target Vaidy A, Forfia P, Mazurek J, Vaidya A. BMJ Case Rep. 2021 Mar 20;14(3): e228358. doi: 10.1136/bcr-2018-228358. Vaidy A BMJ Case Rep 2021 22-03-2021 PMC7986860.
163. 27465384 Effects of matched weight loss from calorie restriction, exercise, or both on cardiovascular disease risk factors: a randomized intervention trial Weiss EP, Albert SG, Reeds DN, Kress KS, McDaniel JL, Klein S, Villareal DT. Am J Clin Nutr. 2016 Sep;104(3):576-86. doi: 10.3945/ajcn.116.131391. Epub 2016 Jul 27. Weiss EP Am J Clin Nutr 2016 29-07-2016 PMC4997297.
164. 28183819 A chronic physical activity treatment in obese rats normalizes the contributions of ET-1 and NO to insulin-mediated posterior cerebral artery vasodilation Olver TD, McDonald MW, Klakotskaia D, Richardson RA, Jasperse JL, Melling CWJ, Schachtman TR, Yang HT, Emter CA, Laughlin MH. J Appl Physiol (1985). 2017 Apr 1;122(4):1040-1050. doi: 10.1152/japplphysiol.00811.2016. Epub 2017 Feb 9. Olver TD J Appl Physiol (1985) 2017 11-02-2017 PMC5407197.
165. 22364114 Pedometer-determined physical activity is linked to low systemic inflammation and low arterial stiffness in Type 2 diabetes Jennersjö P, Ludvigsson J, Länne T, Nystrom FH, Ernerudh J, Östgren CJ. Diabet Med. 2012 Sep;29(9):1119-25. doi: 10.1111/j.1464-5491.2012.03621. x. Jennersjö P Diabet Med 2012 28-02-2012.
166. 31670382 Overcoming exercise barriers: home-based HIT for reducing cardiovascular disease risk in obese individuals McCarty NP, Craighead DH, Freeberg KA. J Physiol. 2020 Jan;598(1):13-14. doi: 10.1113/JP279074. Epub 2019 Dec 3. McCarty NP J Physiol 2020 01-11-2019.
167. 19681467 [Primary prevention of cardiovascular disease] Katić T, Sakić I, Bergovec M. Acta Med Croatica. 2009 Feb;63(1):71-4. Katić T. Acta Med Croatica 2009 18-08-2009.
168. 21317309 Differential vulnerability of skeletal muscle feed arteries to dysfunction in insulin resistance: impact of fiber type and daily activity Bender SB, Newcomer SC, Harold Laughlin M. Am J Physiol Heart Circ Physiol. 2011 Apr;300(4):H1434-41. doi: 10.1152/ajpheart.01093.2010. Epub 2011 Feb 11. Bender SB Am J Physiol Heart Circ Physiol 2011 15-02-2011 PMC3075034.
169. 18949930 [Physical activity in the prevention and treatment of cardiovascular diseases] Mirat J. Acta Med Croatica. 2007;61 Suppl 1:63-7. Mirat J Acta Med Croatica 2007 28-10-2008.
170. 20634354 Daily physical activity enhances reactivity to insulin in skeletal muscle arterioles of hyperphagic Otsuka Long-Evans Tokushima Fatty rats Mikus CR, Rector RS, Arce-Esquivel AA, Libla JL, Booth FW, Ibdah JA, Laughlin MH, Thyfault JP. J Appl Physiol (1985). 2010 Oct;109(4):1203-10. doi: 10.1152/japplphysiol.00064.2010. Epub 2010 Jul 15. Mikus CR J Appl Physiol (1985) 2010 17-07-2010 PMC2963325.
171. 25896283 Role of obesity on cerebral hemodynamics and cardiorespiratory responses in healthy men during repetitive incremental lifting Cavuoto LA, Maikala RV. Eur J Appl Physiol. 2015 Sep;115(9):1905-17. doi: 10.1007/s00421-015-3171-3. Epub 2015 Apr 21. Cavuoto LA Eur J Appl Physiol 2015 22-04-2015.
172. 20889128 Branched-chain amino acid supplementation promotes survival and supports cardiac and skeletal muscle mitochondrial biogenesis in middle-aged mice D'Antona G, Ragni M, Cardile A, Tedesco L, Dossena M, Bruttini F, Caliaro F, Corsetti G, Bottinelli R, Carruba MO, Valerio A, Nisoli E. Cell Metab. 2010 Oct 6;12(4):362-372. doi: 10.1016/j.cmet.2010.08.016. D'Antona G Cell Metab 2010 05-10-2010.
173. 24393423 Step Monitoring to improve ARTERial health (SMARTER) through step count prescription in type 2 diabetes and hypertension: trial design and methods Dasgupta K, Rosenberg E, Daskalopoulou SS; SMARTER collaborators. Cardiovasc Diabetol. 2014 Jan 6; 13:7. doi: 10.1186/1475-2840-13-7. Dasgupta K Cardiovasc Diabetol 2014 08-01-2014 PMC3893520.
174. 18278156 [Physical activity in the prevention of cardiovascular diseases. Epidemiology and mechanisms] Hilberg T. Hamostaseologie. 2008 Feb;28(1-2):9-12, 14-15. Hilberg T Hamostaseologie 2008 19-02-2008.
175. 31261886 Endothelial Dysfunction in Chronic Heart Failure: Assessment, Findings, Significance, and Potential Therapeutic Targets Alem MM. Int J Mol Sci. 2019 Jun 29;20(13):3198. doi: 10.3390/ijms20133198. Alem MM Int J Mol Sci 2019 03-07-2019 PMC6651535.
176. 29932275 Improved brachial artery shear patterns and increased flow-mediated dilatation after low-volume high-intensity interval training in type 2 diabetes Ghardashi Afousi A, Izadi MR, Rakhshan K, Mafi F, Biglari S, Gandomkar Bagheri H. Exp Physiol. 2018 Sep;103(9):1264-1276. doi: 10.1113/EP087005. Epub 2018 Jul 22. Ghardashi Afousi A Exp Physiol 2018 23-06-2018.

**Studies included in review:**

***N*** **=** **3**

2. 31343521 Low-Frequency HIIT Improves Body Composition and Aerobic Capacity in Overweight Men Chin EC, Yu AP, Lai CW, Fong DY, Chan DK, Wong SH, Sun F, Ngai HH, Yung PSH, Siu PM. Med Sci Sports Exerc. 2020 Jan;52(1):56-66. doi: 10.1249/MSS.0000000000002097. Chin EC Med Sci Sports Exerc 2020 26-07-2019.

71. 34816612 Effects of indulgent food snacking, with and without exercise training, on body weight, fat mass, and cardiometabolic risk markers in overweight and obese men Tucker WJ, Jarrett CL, D'Lugos AC, Angadi SS, Gaesser GA. Physiol Rep. 2021 Nov;9(22): e15118. doi: 10.14814/phy2.15118. Tucker WJ Physiol Rep 2021 24-11-2021 PMC8611507.

100. 32577192 The Impact of High-Intensity Interval Training Versus Moderate-Intensity Continuous Training on Carotid Intima-Media Thickness and Ankle-Brachial Index in Middle-Aged Women Farahati S, Hosseini SRA, Moazzami M, Daloee MH, Daloee SH. Int J Prev Med. 2020 Jun 3; 11:62. doi: 10.4103/ijpvm.IJPVM_524_18. e Collection 2020. Farahati S Int J Prev Med 2020 25-06-2020 PMC7297415.

## References

[1] GBD 2015 Obesity Collaborators; Afshin A, Forouzanfar MH, Reitsma MB, Sur P, Estep K, Lee A, et al. Health effects of overweight and obesity in 195 countries over 25 years. N Engl J Med. 2017;377:13–27. 10.1056/NEJMoa1614362 PMC5477817 28604169

[2] Smith KB, Smith MS. Obesity statistics. Prim Care Clin Off Pract. 2016;43:121–35. 10.1016/j.pop.2015.10.001 26896205

[3] Rossi R, Iaccarino D, Nuzzo A, Chiurlia E, Bacco L, Venturelli A, et al. Influence of body mass index on extent of coronary atherosclerosis and cardiac events in a cohort of patients at risk of coronary artery disease. Nutr Metab Cardiovasc Dis. 2011;21:86–93. 19939651 10.1016/j.numecd.2009.09.001

[4] Koenen M, Hill MA, Cohen P, Sowers JR. Obesity, adipose tissue and vascular dysfunction. Circ Res. 2021;128:951–68. 33793327 10.1161/CIRCRESAHA.121.318093 PMC8026272

[5] Klein S, Burke LE, Bray GA, Blair S, Allison DB, Pi-Sunyer X, et al. Clinical implications of obesity with specific focus on cardiovascular disease. Circulation. 2004;110:2952–67. 15509809 10.1161/01.CIR.0000145546.97738.1E

[6] Nanchahal K, Morris JN, Sullivan LM, Wilson PWF. Coronary heart disease risk in men and the epidemic of overweight and obesity. Int J Obes. 2005;29:317–23. 10.1038/sj.ijo.0802877 15597108

[7] Fornoni A, Raij L. Metabolic syndrome and endothelial dysfunction. Curr Hypertens Rep. 2005;7:88–95. 15748531 10.1007/s11906-005-0080-6

[8] Shimokawa H, Satoh K. Vascular function. Arterioscler Thromb Vasc Biol. 2014;34:2359–62. 25147337 10.1161/ATVBAHA.114.304119

[9] Sena CM, Gonçalves L, Seiça R. Methods to evaluate vascular function: a crucial approach towards predictive, preventive, and personalised medicine. EPMA J. 2022;13:209–35. 10.1007/s13167-022-00280-7 PMC9120812 35611340

[10] Green DJ, Smith KJ. Effects of exercise on vascular function, structure, and health in humans. Cold Spring Harb Perspect Med. 2018;8:a029819. 28432115 10.1101/cshperspect.a029819 PMC5880156

[11] Joyner MJ, Green DJ. Exercise protects the cardiovascular system: effects beyond traditional risk factors. J Physiol. 2009;587:5551–8. 19736305 10.1113/jphysiol.2009.179432 PMC2805367

[12] Powers SK, Jackson MJ. Exercise-induced oxidative stress: cellular mechanisms and impact on muscle force production. Physiol Rev. 2008;88:1243–76. 18923182 10.1152/physrev.00031.2007 PMC2909187

[13] Holloszy JO. Regulation by exercise of skeletal muscle content of mitochondria and GLUT4. J Physiol Pharm. 2008;59:5–18. 19258654

[14] Prior BM, Yang HT, Terjung RL. What makes vessels grow with exercise training? J Appl Physiol. 2004;97:1119–28. 15333630 10.1152/japplphysiol.00035.2004

[15] Mezzani A, Hamm LF, Jones AM, McBride PE, Moholdt T, Stone JA, et al. Aerobic exercise intensity assessment and prescription in cardiac rehabilitation: a joint position statement of the European Association for Cardiovascular Prevention and Rehabilitation, the American Association of Cardiovascular and Pulmonary Rehabilitation and the Canadian Association of Cardiac Rehabilitation. Eur J Prev Cardiol. 2013;20:442–67. 23104970 10.1177/2047487312460484

[16] Atakan MM, Li Y, Koşar ŞN, Turnagöl HH, Yan X. Evidence-based effects of high-intensity interval training on exercise capacity and health: a review with historical perspective. Int J Environ Res Public Health. 2021;18:7201. 10.3390/ijerph18137201 PMC8294064 34281138

[17] D’Amuri A, Sanz JM, Capatti E, Di Vece F, Vaccari F, Lazzer S, et al. Effectiveness of high-intensity interval training for weight loss in adults with obesity: a randomised controlled non-inferiority trial. BMJ Open Sport Exerc Med. 2021;7:e001021. 34367654 10.1136/bmjsem-2020-001021 PMC8292807

[18] Mendelson M, Chacaroun S, Baillieul S, Doutreleau S, Guinot M, Wuyam B, et al. Effects of high intensity interval training on sustained reduction in cardiometabolic risk associated with overweight/obesity. A randomized trial. J Exerc Sci Fit. 2022;20:172–81. 35401768 10.1016/j.jesf.2022.03.001 PMC8956941

[19] Sawyer BJ, Tucker WJ, Bhammar DM, Ryder JR, Sweazea KL, Gaesser GA. Effects of high-intensity interval training and moderate-intensity continuous training on endothelial function and cardiometabolic risk markers in obese adults. J Appl Physiol. 2016;121:279–88. 27255523 10.1152/japplphysiol.00024.2016 PMC4967258

[20] Weston KS, Wisløff U, Coombes JS. High-intensity interval training in patients with lifestyle-induced cardiometabolic disease: a systematic review and meta-analysis. Br J Sports Med. 2014;48:1227–34. 24144531 10.1136/bjsports-2013-092576

[21] Ramos JS, Dalleck LC, Tjonna AE, Beetham KS, Coombes JS. The impact of high-intensity interval training versus moderate-intensity continuous training on vascular function: a systematic review and meta-analysis. Sports Med. 2015;45:679–92. 10.1007/s40279-015-0321-z 25771785

[22] Chin EC, Yu AP, Lai CW, Fong DY, Chan DK, Wong SH, et al. Low-frequency HIIT improves body composition and aerobic capacity in overweight men. Med Sci Sports Exerc. 2020;52:56–66. 31343521 10.1249/MSS.0000000000002097

[23] Shenouda N, Gillen JB, Gibala MJ, Macdonald MJ, Macdonald M. Changes in brachial artery endothelial function and resting diameter with moderate-intensity continuous but not sprint interval training in sedentary men. J Appl Physiol. 2017;123:773–80. 28546466 10.1152/japplphysiol.00058.2017 PMC5668454

[24] Khalafi M, Sakhaei MH, Kazeminasab F, Symonds ME, Rosenkranz SK. The impact of high-intensity interval training on vascular function in adults: a systematic review and meta-analysis. Front Cardiovasc Med. 2022;9:1046560. 10.3389/fcvm.2022.1046560 PMC9713318 36465439

[25] Sabouri M, Amirshaghaghi F, Hesari MM. High-intensity interval training improves the vascular endothelial function comparing moderate-intensity interval training in overweight or obese adults: a meta-analysis. Clin Nutr ESPEN. 2023;53:100–6. 36657899 10.1016/j.clnesp.2022.11.023

[26] Way KL, Sultana RN, Sabag A, Baker MK, Johnson NA. The effect of high intensity interval training versus moderate intensity continuous training on arterial stiffness and 24 h blood pressure responses: a systematic review and meta-analysis. J Sci Med Sport. 2019;22:385–91. 10.1016/j.jsams.2018.09.228 30803498

[27] Page MJ, McKenzie JE, Bossuyt PM, Boutron I, Hoffmann TC, Mulrow CD, et al. The PRISMA 2020 statement: an updated guideline for reporting systematic reviews. PLoS Med. 2021;18:e1003583. 33780438 10.1371/journal.pmed.1003583 PMC8007028

[28] Higgins JPT, Thomas J, Chandler J, Cumpston M, Li T, Page MJ, et al. Cochrane handbook for systematic reviews of interventions version 6.2. Cochrane; 2021. Available from www.training.cochrane.org/handbook.

[29] Shepherd SO, Wilson OJ, Taylor AS, Thøgersen-Ntoumani C, Adlan AM, Wagenmakers AJM, et al. Low-volume high-intensity interval training in a gym setting improves cardio-metabolic and psychological health. PLoS ONE. 2015;10:e0139056. 26402859 10.1371/journal.pone.0139056 PMC4581708

[30] Cheema BS, Davies TB, Stewart M, Papalia S, Atlantis E. The feasibility and effectiveness of high-intensity boxing training versus moderate-intensity brisk walking in adults with abdominal obesity: a pilot study. BMC Sports Sci Med Rehabil. 2015;7:3. 25973207 10.1186/2052-1847-7-3 PMC4429464

[31] Cocks M, Shaw CS, Shepherd SO, Fisher JP, Ranasinghe A, Barker TA, et al. Sprint interval and moderate-intensity continuous training have equal benefits on aerobic capacity, insulin sensitivity, muscle capillarisation and endothelial eNOS/NAD(P)Hoxidase protein ratio in obese men. J Physiol. 2016;594:2307–21. 25645978 10.1113/jphysiol.2014.285254 PMC4933110

[32] Schjerve IE, Tyldum GA, Tjønna AE, Stølen T, Loennechen JP, Hansen HEM, et al. Both aerobic endurance and strength training programmes improve cardiovascular health in obese adults. Clin Sci. 2008;115:283–93. 10.1042/CS20070332 18338980

[33] Shi W, Chen J, He Y, Su P, Wang M, Li X, et al. The effects of high-intensity interval training and moderate-intensity continuous training on visceral fat and carotid hemodynamics parameters in obese adults. J Exerc Sci Fit. 2022;20:355–65. 36186829 10.1016/j.jesf.2022.09.001 PMC9486563

[34] Bækkerud FH, Solberg F, Leinan IM, WislØff U, Karlsen T, Rognmo Ø. Comparison of three popular exercise modalities on VO2max in overweight and obese. Med Sci Sports Exerc. 2016;48:491–8. 26440134 10.1249/MSS.0000000000000777

[35] Tucker WJ, Jarrett CL, D’Lugos AC, Angadi SS, Gaesser GA. Effects of indulgent food snacking, with and without exercise training, on body weight, fat mass, and cardiometabolic risk markers in overweight and obese men. Physiol Rep. 2021;9:e15118. 34816612 10.14814/phy2.15118 PMC8611507

[36] Farahati S, Attarzadeh Hosseini S, Moazzami M, Daloee M, Daloee S. The impact of high-intensity interval training versus moderate-intensity continuous training on carotid intima-media thickness and ankle-brachial index in middle-aged women. Int J Prev Med. 2020;11:62. 32577192 10.4103/ijpvm.IJPVM_524_18 PMC7297415

[37] van Sloten TT, Henry RMA, Dekker JM, Nijpels G, Unger T, Schram MT, et al. Endothelial dysfunction plays a key role in increasing cardiovascular risk in type 2 diabetes. Hypertension. 2014;64:1299–305. 25225211 10.1161/HYPERTENSIONAHA.114.04221

[38] Di Francescomarino S, Sciartilli A, Di Valerio V, Di Baldassarre A, Gallina S. The effect of physical exercise on endothelial function. Sports Med. 2009;39:797–812. 19757859 10.2165/11317750-000000000-00000

[39] Teixeira-Lemos E, Nunes S, Teixeira F, Reis F. Regular physical exercise training assists in preventing type 2 diabetes development: focus on its antioxidant and anti-inflammatory properties. Cardiovasc Diabetol. 2011;10:12. 21276212 10.1186/1475-2840-10-12 PMC3041659

[40] Wisløff U, Støylen A, Loennechen JP, Bruvold M, Rognmo Ø, Haram PM, et al. Superior cardiovascular effect of aerobic interval training versus moderate continuous training in heart failure patients. Circulation. 2007;115:3086–94. 17548726 10.1161/CIRCULATIONAHA.106.675041

[41] Tjønna AE, Lee SJ, Rognmo Ø, Stølen TO, Bye A, Haram PM, et al. Aerobic interval training versus continuous moderate exercise as a treatment for the metabolic syndrome. Circulation 2008;118:346–54. 18606913 10.1161/CIRCULATIONAHA.108.772822 PMC2777731

[42] Zou C, Jiao Y, Li X, Zheng C, Chen M, Hu C. Role of ultrasonography in the evaluation of correlation between strain and elasticity of common carotid artery in patients with diabetic nephropathy. Int J Clin Exp Med. 2015;8:17765–72. 26770367 PMC4694267

[43] Ashor AW, Lara J, Siervo M, Celis-Morales C, Mathers JC. Effects of exercise modalities on arterial stiffness and wave reflection: a systematic review and meta-analysis of randomized controlled trials. PLoS ONE. 2014;9:e110034. 25333969 10.1371/journal.pone.0110034 PMC4198209

[44] Huang C, Wang J, Deng S, She Q, Wu L. The effects of aerobic endurance exercise on pulse wave velocity and intima media thickness in adults: a systematic review and meta‐analysis. Scand J Med Sci Sports. 2016;26:478–87. 26059748 10.1111/sms.12495

[45] Wewege M, van den Berg R, Ward RE, Keech A. The effects of high‐intensity interval training vs. moderate‐intensity continuous training on body composition in overweight and obese adults: a systematic review and meta‐analysis. Obes Rev. 2017;18:635–46. 28401638 10.1111/obr.12532

[46] Sultana RN, Sabag A, Keating SE, Johnson NA. The effect of low-volume high-intensity interval training on body composition and cardiorespiratory fitness: a systematic review and meta-analysis. Sports Med. 2019;49:1687–721. 31401727 10.1007/s40279-019-01167-w

[47] Wilson OWA, Walters SR, Naylor ME, Clarke JC. Physical activity and associated constraints following the transition from high school to university. Recreat Sports J. 2021;45:52–60.

[48] McGuinness LA, Higgins JPT. Risk-of-bias VISualization (robvis): An R package and Shiny web app for visualizing risk-of-bias assessments. Res Syn Meth. 2020;1–7. 10.1002/jrsm.1411. 10.1002/jrsm.1411 32336025

